# Critical role of reproductive tract microbiota and derived metabolites in inflammation, tumor immunity, and tumorigenesis of gynecological cancers: a narrative review

**DOI:** 10.3389/fimmu.2026.1734792

**Published:** 2026-03-04

**Authors:** Hong Chen, Ge Lou, Fanling Meng, Yang Zhang, Hongying Kuang, Dongxia Yang

**Affiliations:** 1 Graduate School, The Second Affiliated Hospital of Heilongjiang University of Chinese Medicine, Harbin, Heilongjiang, China; 2 Department of Gynecology, Harbin Medical University Cancer Hospital, Harbin, Heilongjiang, China; 3 Graduate School, Heilongjiang University of Chinese Medicine, Harbin, Heilongjiang, China; 4 Department of Internal Medicine, The First Affiliated Hospital of Heilongjiang University of Chinese Medicine, Harbin, Heilongjiang, China; 5 Department of Gynecology, The First Affiliated Hospital of Heilongjiang University of Chinese Medicine, Harbin, Heilongjiang, China; 6 Department of Gynecology, The Second Affiliated Hospital of Heilongjiang University of Chinese Medicine, Harbin, Heilongjiang, China

**Keywords:** gynecological cancers, Microbiome, microbial metabolites, immunotherapy, chemotherapy resistance

## Abstract

Gynecological malignancies, including ovarian, cervical, and endometrial cancers, present significant clinical challenges due to the epidemiological complexity and limitations in current therapeutic strategies. Emerging evidence highlights the critical role of the microbiome and its metabolites in modulating tumor initiation, progression, and treatment responses. This review explores the intricate mechanisms through which gut and reproductive tract microbiota influence gynecological cancers via immune regulation, metabolic reprogramming, and epigenetic modifications. Key microbial metabolites, such as short-chain fatty acids, bile acids, and estrogen-metabolizing intermediates, serve as molecular bridges in host-microbe communication, impacting chemotherapy resistance and immunotherapy efficacy. Furthermore, we discuss the translational potential of microbiome-targeted interventions, including probiotics, fecal microbiota transplantation, and precision microbial therapies, as innovative approaches for diagnosis, prognosis, and treatment. Understanding the microbiota-reproductive axis offers novel insights into overcoming therapeutic resistance and improving patient outcomes in gynecologic oncology.

## 1 Introduction

Ovarian, cervical, and endometrial carcinomas represent the principal malignancies of the female reproductive tract. The complex epidemiology of these diseases, alongside the exigencies of the prevention and clinical management, continues to present formidable challenges to modern oncology (1–3). Ovarian carcinoma is frequently diagnosed at an advanced stage, a phenomenon attributable to its insidious onset and the current limitations in early detection modalities. This diagnostic latency is a primary driver of its status as the most lethal gynecological malignancy (4, 5). In contrast, the widespread implementation of human papillomavirus (HPV) vaccines and the promotion of systematic screening have contributed to a declining incidence of cervical cancer in certain regions. Nevertheless, in areas with limited medical resources, the burden of the disease remains substantial (6, 7). Endometrial cancer is intimately linked to a range of factors including obesity, metabolic disorders, and endocrine imbalances, with its global incidence experiencing a steady rise in recent years, underscoring the need for heightened awareness and attention (8). Current therapeutic paradigms for gynecological malignancies predominantly rely on surgical resection, chemotherapy, and radiotherapy, alongside the increasingly pivotal role of immunotherapy. However, the clinical success of these modalities is frequently compromised by significant limitations. Specifically, the acquisition of resistance to chemotherapy and radiotherapy often precipitates disease progression and recurrence. Furthermore, the efficacy of immunotherapy exhibits profound heterogeneity across patient cohorts, being severely attenuated by the immunosuppressive landscape of the tumor microenvironment (TME) (9, 10). Consequently, the pursuit of innovative therapeutic strategies aimed at reversing resistance mechanisms, reconstituting the immune microenvironment, and ultimately enhancing survival outcomes for patients has emerged as a pivotal focus in the realm of gynecological cancers research. In the quest to unravel solutions to complex biological questions, the human second genome—the microbiome—has increasingly captured the attention of the scientific community as an emergent microbial organ. Within mammals, a highly diverse and dynamically active community of microorganisms exists, whose interactions and co-evolution with the hosts commence at birth and profoundly influence the host’s physiological functions throughout adulthood (11, 12). Research evidence has showed that a mutualistic symbiotic relationship has developed over long-term co-evolution between the human microbiome and its host. Harboring tens of thousands of microbial genomes and over 80 million genes, this ecosystem offers metabolic capabilities that vastly exceed those of the host (13). Concurrently, the host organism has evolved sophisticated regulatory mechanisms to integrate conserved metabolic signals, microbiome recognition systems, and immune response pathways, thereby preserving homeostasis within the microbiota-dominated environment. This close symbiotic relationship positions the microbiome as a pivotal entity in the regulation of the host’s physiological and pathological states, leading to its compelling designation as the second genome or microbial organ (11, 14, 15).

**Figure 1:**
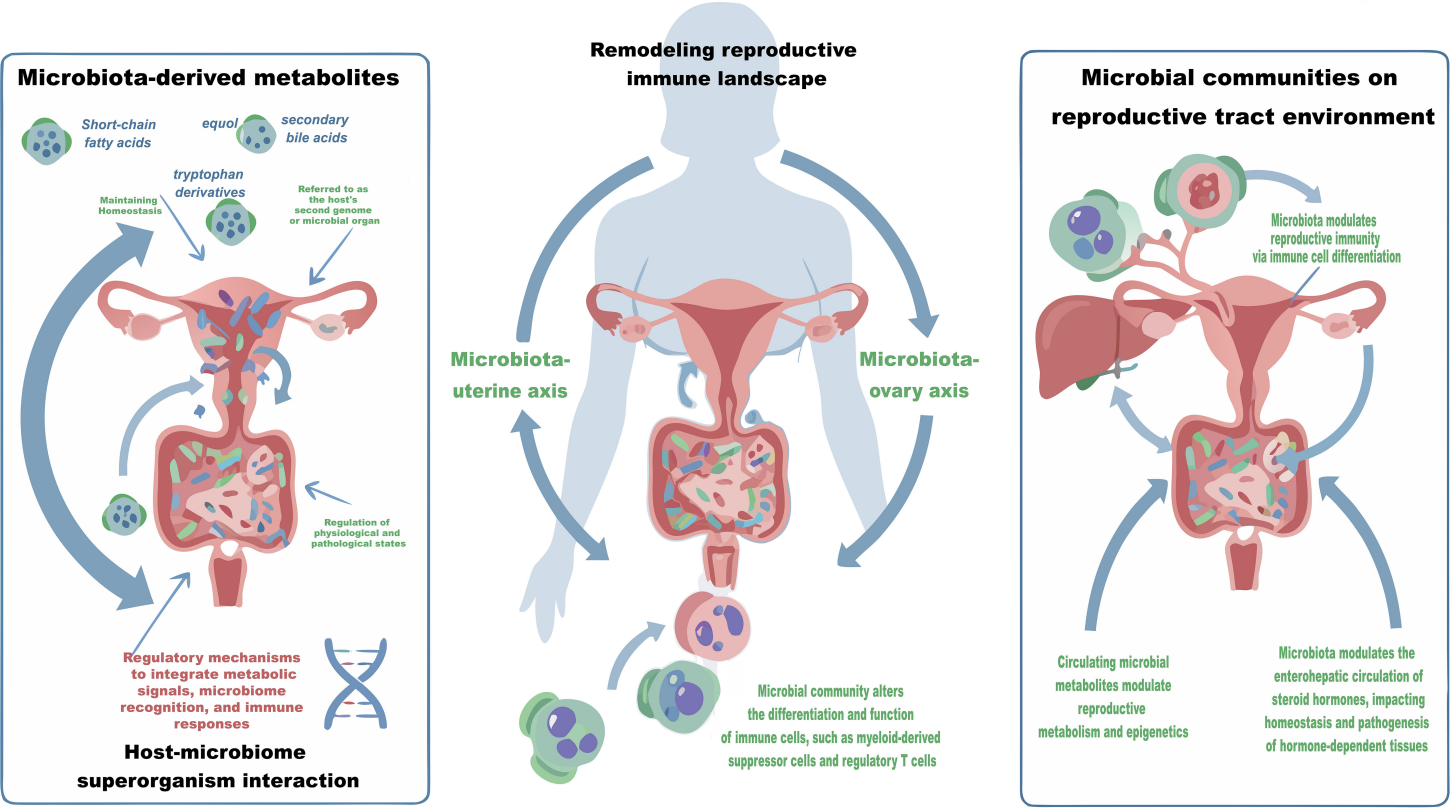
The microbiota-reproductive tract axis in female reproductive health and gynecological malignancies. The complex bidirectional crosstalk exists between the host and its microbiome, with the microbiome-uterus and microbiome-ovary axes playing integral roles in regulating reproductive pathophysiology. As a symbiotic super-organism, the host integrates microbial signals to maintain homeostasis through several key mechanisms. Microbiome-derived metabolites enter the systemic circulation via the portal vein to modulate cellular metabolism and epigenetic landscapes in distal reproductive organs. Concurrently, the microbiota reshapes the local immune microenvironment by altering the differentiation and function of immune cells. Furthermore, through the regulation of enterohepatic circulation and metabolism of estrogens and other steroid hormones, the microbiome influences the susceptibility of hormone-dependent tissues to pathological changes.

It is noteworthy that the microbiome, as a key regulatory hub, has roles that extend beyond the traditional digestive system. It mediates systemic regulation of distant organ functions through the microbiota -organ axes (microbiota -brain axis, microbiota -liver axis) (16). As shown in **Figure 1**, the regulatory function relies on a complex and highly coordinated bidirectional communication network between the host and the microbial community, with mechanisms primarily reflected in the following three aspects: Firstly, microbes significantly expand the host’s metabolic potential and participate in various physiological processes, including dietary fiber fermentation, protein degradation, and vitamin synthesis. Secondly, microbes provide the host with colonization resistance through niche competition and immune modulation, playing a crucial role in shaping the development and functional balance of the immune system. Importantly, microbial-derived metabolites can function as signaling molecules that enter the circulatory system and regulate the physiological state of distant organs via nervous, endocrine, and immune pathways. In response, the host has evolved a multi-layered regulatory system that integrates pattern recognition, metabolic sensing, and immune responses to maintain the dynamic balance of the symbiotic system (11, 17–19). The host-microbiome superorganism interaction model not only deepens our understanding of physiological homeostasis regulation mechanisms but also provides new perspectives for understanding clinical issues such as disease susceptibility differences and drug response heterogeneity, particularly showing great translational medical potential in cancer prevention and treatment.

A burgeoning body of evidence is progressively unraveling the intricate crosstalk between the microbiome and female reproductive physiology. Particular attention has been focused on the regulatory networks governing the microbiota-organ axis. Accumulating evidence demonstrates a dynamic, bidirectional crosstalk between the gut/vaginal microbiota and female reproductive organs, substantiating the concept of the ‘microbiota-reproductive tract axis.’ This axis can be further stratified into anatomically distinct pathways, including the microbiota-uterus and microbiota-ovary axes. Collectively, these signaling cascades provide a mechanistic framework for understanding the microbial modulation of female reproductive health (20–23). From a mechanistic perspective, microbiota plays a crucial role in both maintaining and disrupting the homeostasis of the reproductive tract microenvironment through multiple intricate pathways. Firstly, metabolites produced by microbiota, short-chain fatty acids (SCFAs), secondary bile acids (SBAs), and equol, enter systemic circulation via portal vein, directly influencing distal reproductive organs and modulating cellular metabolism and epigenetic processes. Secondly, microbiota significantly impacts the differentiation and functionality of immune cells, including myeloid-derived suppressor cells (MDSCs) and regulatory T cells (Tregs), thereby reshaping the local immune microenvironment of reproductive tract. Thirdly, microbiota actively regulates the enterohepatic circulation and metabolism of steroid hormones, such as estrogen, which in turn governs the physiological and pathological states of hormone-dependent tissues. Collectively, these mechanisms establish a robust molecular foundation for understanding the influence of microbiota on the onset and progression of gynecological cancers (11, 14, 17, 21, 22, 24, 25). Elucidating the mechanistic underpinnings of gynecological malignancies is of paramount clinical importance. Currently, chemotherapy resistance and the immunosuppressive nature of the TME represent formidable therapeutic hurdles—both of which are intrinsically tethered to the functional dynamics of the microbiota. Emerging evidences have indicated that specific bacterial taxa dictate immunotherapeutic efficacy by orchestrating immune cell infiltration and function within the TME. Concurrently, microbiota-derived metabolites have been shown to sensitize tumor cells to platinum-based regimens, largely through epigenetic reprogramming (26–28). These pivotal findings furnish valuable insights that can inform the optimization of clinical treatment strategies. This narrative review critically elucidates the pivotal role of the microbiome-reproductive axis across three major gynecological malignancies: ovarian, cervical, and endometrial cancers. Through a comprehensive preliminary analysis of information gathered from PubMed, Scholar, Embase, and Scopus, utilizing search keywords such as “microbiota,” “microbiome,” “gynecological cancers,” “ovarian cancer,” “cervical cancer,” “endometrial cancer,” “metabolites,” and “immunotherapy,” we dissect the multifaceted influence of the microbiota and its derived metabolites on tumor initiation, disease progression, and therapeutic outcomes. Our rigorous filtering prioritized English-language articles, including original researches, clinical trials, and relevant reviews, to ensure a robust foundation for examining the regulatory roles of the microbiome in host immunity, metabolic homeostasis, and epigenetic reprogramming. Furthermore, this review critically evaluates the translational avenues for microbiome-based diagnostic biomarkers and explores the current challenges and significant potential of integrating microbiome interventions as novel therapeutic adjuvants, aiming to provide a deeper understanding for overcoming therapeutic resistance and improving patient outcomes in gynecologic oncology.

## 2 Microbial immunomodulation in the female reproductive tract

The host immune system must navigate a complex physiological dichotomy: sustaining a state of robust tolerance toward commensal microbiota and semi-allogeneic fetal antigens, while concurrently retaining the potency to execute cytotoxic defense against pathogens and malignant transformation. This delicate equilibrium is not autonomously regulated by the host but is profoundly shaped by the microbiota via a complex chemical communication network that extends beyond the local microbiota ecosystem. In this context, microbial metabolites serve as critical immunological ligands rather than mere metabolic byproducts. These bioactive compounds are primarily derived from the anaerobic fermentation and biotransformation of dietary indigestible components (dietary fibers, aromatic amino acids) and endogenous host compounds (primary bile acids). Key functional classes exert the biological functions through diverse signaling pathways: SCFAs (acetate, propionate, butyrate) not only inhibit histone deacetylases (HDACs) to regulate gene transcription via epigenetic mechanisms (11, 14, 17, 22, 24, 25, 29), but also activate G protein-coupled receptors (GPCRs), directly driving immune cell differentiation and barrier integrity (30, 31). SBAs modulate inflammatory responses by activating nuclear receptors like the farnesoid X receptor (FXR) (32); and structural patterns like lipopolysaccharide (LPS) engage innate sensors. Furthermore, the microbiota encompasses a specialized functional aggregate termed the ‘estrobolome,’ which is integral to estrogen homeostasis. Through specific enzymatic activities, this module facilitates the deconjugation of sequestered estrogens into the biologically active forms. Consequently, this reactivation heightens hormonal bioavailability, thereby exerting a profound regulatory influence on hormone-dependent physiological cascades and the local immune microenvironment (33–36). By engaging these specific host receptors and modifying epigenetic landscapes, these metabolites collectively orchestrate the crosstalk between innate sensing, adaptive immunity, and the inflammatory microenvironment (37, 38).

### 2.1 Innate immune sensing and the threshold of inflammation

**Figure 2:**
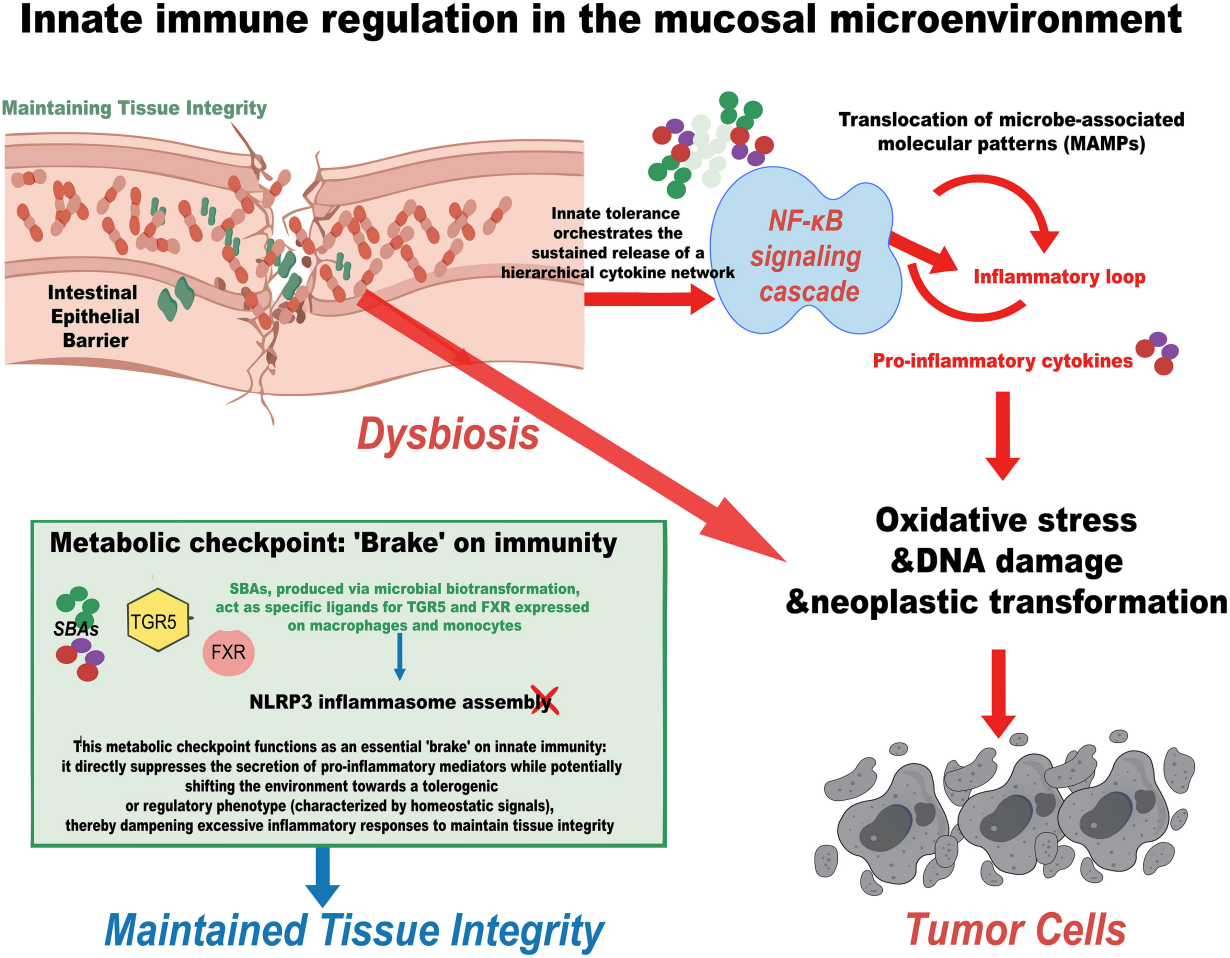
Innate immune sensing mechanisms regulating the threshold of mucosal inflammation and tumorigenesis. Under dysbiotic conditions, mucosal barrier compromise facilitates the translocation of microbe-associated molecular patterns (MAMPs), such as LPS. Engagement of TLR4 by LPS triggers the NF-κB signaling cascade in innate immune cells, orchestrating the release of pro-inflammatory cytokines and chemokines. This inflammatory loop drives oxidative stress, DNA damage, and myeloid cell recruitment, promoting neoplastic transformation. Conversely, secondary bile acids (SBAs) produced via microbial biotransformation activate the metabolic sensors TGR5 and FXR. This signaling pathway acts as an innate immune "brake" by suppressing NLRP3 inflammasome assembly and NF-κB activity, thereby dampening inflammation and maintaining tissue integrity.

The innate immune system utilizes a network of pattern recognition receptors (PRRs) to constantly survey the mucosal microenvironment. The balance between pro-inflammatory activation and anti-inflammatory dampening is tightly modulated by microbial ligands to prevent the chronic inflammation that fuels tumorigenesis. Under homeostatic conditions, mucosal barrier integrity sequesters microbial ligands from the submucosal immune system. Dysbiosis compromises this segregation, facilitating the translocation of microbe-associated molecular patterns (MAMPs), specifically LPS. The engagement of toll-like receptor 4 (TLR4) by LPS initiates a potent nuclear factor (NF)-κB signaling cascade in innate immune cells. This breach of innate tolerance orchestrates the sustained release of a hierarchical cytokine network. Pro-inflammatory cytokines, such as tumor necrosis factor-α (TNF-α), interleukin (IL)-6, and IL-1β are upregulated, driving oxidative stress, DNA damage, and neoplastic transformation (26–28, 37–39). Simultaneously, the signaling triggers the release of chemotactic cytokines like C-X-C motif ligand (CXCL)-8 and C-C motif ligand 2 (CCL2), which actively recruit additional myeloid cells to the tumor site, perpetuating the inflammatory loop. Conversely, the innate immune system possesses negative regulatory mechanisms driven by metabolic sensors. SBAs, produced via microbial biotransformation, act as specific ligands for the Takeda G protein-coupled receptor 5 (TGR5) and FXR expressed on macrophages and monocytes. The activation of TGR5 signaling effectively suppresses the NOD-like receptor family, pyrin domain containing 3 (NLRP3) inflammasome assembly and inhibits NF-κB transcriptional activity. This metabolic checkpoint functions as an essential ‘brake’ on innate immunity: it directly suppresses the secretion of pro-inflammatory mediators while potentially shifting the environment towards a tolerogenic or regulatory phenotype (characterized by homeostatic signals), thereby dampening excessive inflammatory responses to maintain tissue integrity (**Figure 2**) (31, 32, 40–42).

### 2.2 Adaptive immunity: epigenetic and metabolic control of T-cell fate

The plasticity of T lymphocytes—specifically the dynamic equilibrium between immunosuppressive Tregs and cytotoxic effector cells—is a cornerstone of adaptive immunity. This decision-making process is governed by the epigenetic and metabolic environment shaped by the microbiota.

#### 2.2.1 Epigenetic stabilization of tolerance

The preservation of mucosal homeostasis is fundamentally underpinned by the differentiation of forkhead box protein P3 (Foxp3) ^+^ Tregs. It is imperative to delineate this state of physiological tolerance—essential for averting autoimmunity and excessive inflammation—from the pathological immunosuppression that pervades the TME. Mechanistically, SCFAs, particularly butyrate, function as HDACs inhibitors. By facilitating histone hyperacetylation at the *Foxp3* locus, butyrate epigenetically stabilizes the Treg lineage and amplifies the secretion of the anti-inflammatory cytokine IL-10. This molecular orchestration concurrently mitigates NF-κB-driven inflammation and safeguards against autoimmune pathology (29–31, 43, 44).

#### 2.2.2 Enhancement of cytotoxic surveillance

Crucially, microbial metabolites do not merely induce suppression; they also bolster anti-tumor immunity. Through distinct epigenetic reprogramming pathways, SCFAs have been shown to enhance the metabolic fitness, cytotoxicity, and memory function of CD8^+^ T cells, thereby strengthening long-term immune surveillance (45).

#### 2.2.3 Modulation of Th17/Treg balance

Additionally, SCFAs signaling via G-protein-coupled receptor (GPR)-43 on neutrophils and T cells further fine-tunes immune recruitment and cytokine profiles (31). The integrity of the adaptive immune barrier, particularly the function of intraepithelial lymphocytes (IELs), is modulated by ligand availability for the Aryl Hydrocarbon Receptor (AhR). Microbial derivatives of tryptophan (indole compounds) act as AhR agonists, which are crucial for stabilizing the Th17/Treg balance and reinforcing the intestinal and vaginal barriers against aberrant adaptive activation (46–48).

### 2.3 Metabolic enforcement of the immunosuppressive microenvironment

In stark contrast to physiological immune tolerance, which functions as a homeostatic safeguard for mucosal integrity, the immunosuppression engineered by malignancies represents a pathological maladaptation. Malignant cells exploit metabolic reprogramming to construct a nutrient-deprived milieu that abrogates effective antitumor immunity—a phenomenon frequently exacerbated by dysbiotic microbial activities. This metabolic competition imposes a rigorous checkpoint on infiltrating lymphocytes, whose cytotoxic efficacy is contingent upon the availability of essential amino acids. Within the inflammatory context of the TME, the upregulation of Indoleamine 2,3-dioxygenase (IDO) catalyzes the extensive catabolism of tryptophan into kynurenine. The microbiota amplifies the axis, either by directly metabolizing tryptophan or by signaling to perpetuate IDO expression. Consequently, a metabolic ‘double hit’ is inflicted upon adaptive immunity: the acute depletion of tryptophan enforces nutritional starvation, precipitating T-cell anergy, while the accumulation of kynurenine serves as an immunosuppressive oncometabolite. Upon binding to the AhR, kynurenine promotes the polarization of T cells toward a Tregs, thereby rendering the TME an immunologically privileged niche (49). Furthermore, the lytic function of immune effector cells is compromised by the accumulation of polyamines (e.g., putrescine, spermidine). Dysregulated polyamine metabolism, often fueled by microbial synthesis in the gut or local tissues, serves a dual tumorigenic role: it provides essential substrates for rapid tumor cell proliferation while acting as a metabolic brake on immune cells. High polyamine levels have been shown to inhibit the secretion of cytotoxic granules (perforin/granzyme) by CD8^+^ T cells and NK cells, thereby shielding the tumor from immune-mediated destruction (50).

### 2.4 The endocrine-immune axis: hormonal regulation of immunity

The immune system is sexually dimorphic and highly sensitive to steroid hormones, with estrogen receptors (ERs) widely expressed on lymphocytes and macrophages. The microbiota acts as a regulator of this endocrine-immune axis through the “estrobolome”—bacterial populations capable of deconjugating estrogens via β-glucuronidase. Dysbiosis-induced hyperactivity of β-glucuronidase precipitates systemic hyperestrogenemia, which functions as a potent extrinsic regulator of immune plasticity. Mechanistically, ligation of ERs on T cells skews polarization kinetics: it downregulates the Th1 master transcription factor T-bet (thereby dampening interferon-γ (IFN-γ) -mediated cytotoxicity) while reciprocally upregulating GATA3 and Foxp3. This hormonally enforced deviation toward a Th2 or regulatory phenotype constructs an immunosuppressive niche that fundamentally compromises host anti-tumor defense mechanisms (33–36, 51, 52).

### 2.5 Mucosal barrier immunity in reproductive tract

In distinct contrast to the phylogenetic diversity characterizing the gastrointestinal tract, the vaginal mucosa has evolved a unique ecological paradigm predicated on low diversity and high abundance. As delineated in the seminal work by Ravel et al., this ecosystem is typified by the exclusionary dominance of *Lactobacillus* species—particularly *L. crispatus*—which define the healthy Community State Type (CST) I of the reproductive tract. This state of ‘Physiological Immunity’ is biochemically anchored in the metabolism of host-derived glycogen. Through anaerobic fermentation, commensal l*actobacilli* generate high concentrations of lactic acid, sustaining a strictly acidic milieu (pH < 4.5) that serves as a potent physicochemical checkpoint against pathogen colonization. Synergistically, this defensive shield is fortified by the secretion of bioactive effectors, including bacteriocins and hydrogen peroxide, which directly compromise the viability of invading microbes. However, this microbial landscape is far from immutable; recent longitudinal investigations have revealed that the vaginal microbiome exhibits significant temporal plasticity, driven by host physiological variables such as the hormonal fluctuations of the menstrual cycle and fertility status. The collapse of the *lactobacilli-*mediated surveillance triggers a cascade of mucosal compromise: the loss of inhibitory metabolites invites an influx of inflammatory infiltrates, establishing a chronic pro-inflammatory microenvironment that promotes genomic instability and accelerates the trajectory toward carcinogenic transformation (53–56).

## 3 Regulatory roles of microbiota and metabolites on reproductive tract and tumor progression

The microbiota and its host form the largest co-ecological system in the body, crucial for regulating growth, metabolism, and immune adaptations through gene-environment interactions. Dysbiosis—an imbalance in the system—can trigger various diseases by reducing microbial diversity, destabilizing ecology, and promoting pathogen overgrowth, which disrupts immune-metabolic networks (57–59).

### 3.1 Regulating inflammation: double-edged sword effect of microbiota

The maintenance of microbial homeostasis is critical for preserving immunological equilibrium. This is largely because inflammatory mediators within the microenvironment facilitate the recruitment of immune cells, thereby modulating local tissue dynamics (43, 59). In the landscape of chronic inflammatory diseases, extensive research has firmly established that aberrant immune responses—triggered by either autoantigens or environmental stimuli—constitute a foundational pathogenic mechanism. A converging body of evidence underscores that the disruption of the immunological homeostasis does not merely alter immune surveillance; it perpetuates sustained pro-inflammatory signaling cascades. Consequently, this persistent inflammatory milieu emerges as a pivotal driver of oncogenesis, orchestrating the complex biological processes underlying tumor initiation and malignant progression (60, 61). The microbiota acts as a critical regulator of immune homeostasis, characterized by a distinct dichotomy: it is essential for host defense yet capable of driving pathological inflammation when dysregulated (61–63). This bidirectional regulatory axis operates across both local tissue microenvironments and systemic physiological scales, orchestrating a delicate homeostatic equilibrium between pro-inflammatory/pro-tumorigenic drives and immune surveillance. This balance is maintained through a repertoire of multifaceted molecular mechanisms, ranging from metabolite sensing to epigenetic remodeling. Ultimately, the functional impact of the microbiota is dictated by a tripartite intersection: the taxonomic composition of the community, its metabolic output, and the host’s underlying physiological status. Consequently, the perturbation of any component within the intricate nexus may precipitate a collapse in immune homeostasis, thereby creating a permissive landscape for disease progression.

#### 3.1.1 Regulation of immune cells by microbiota

**Figure 3:**
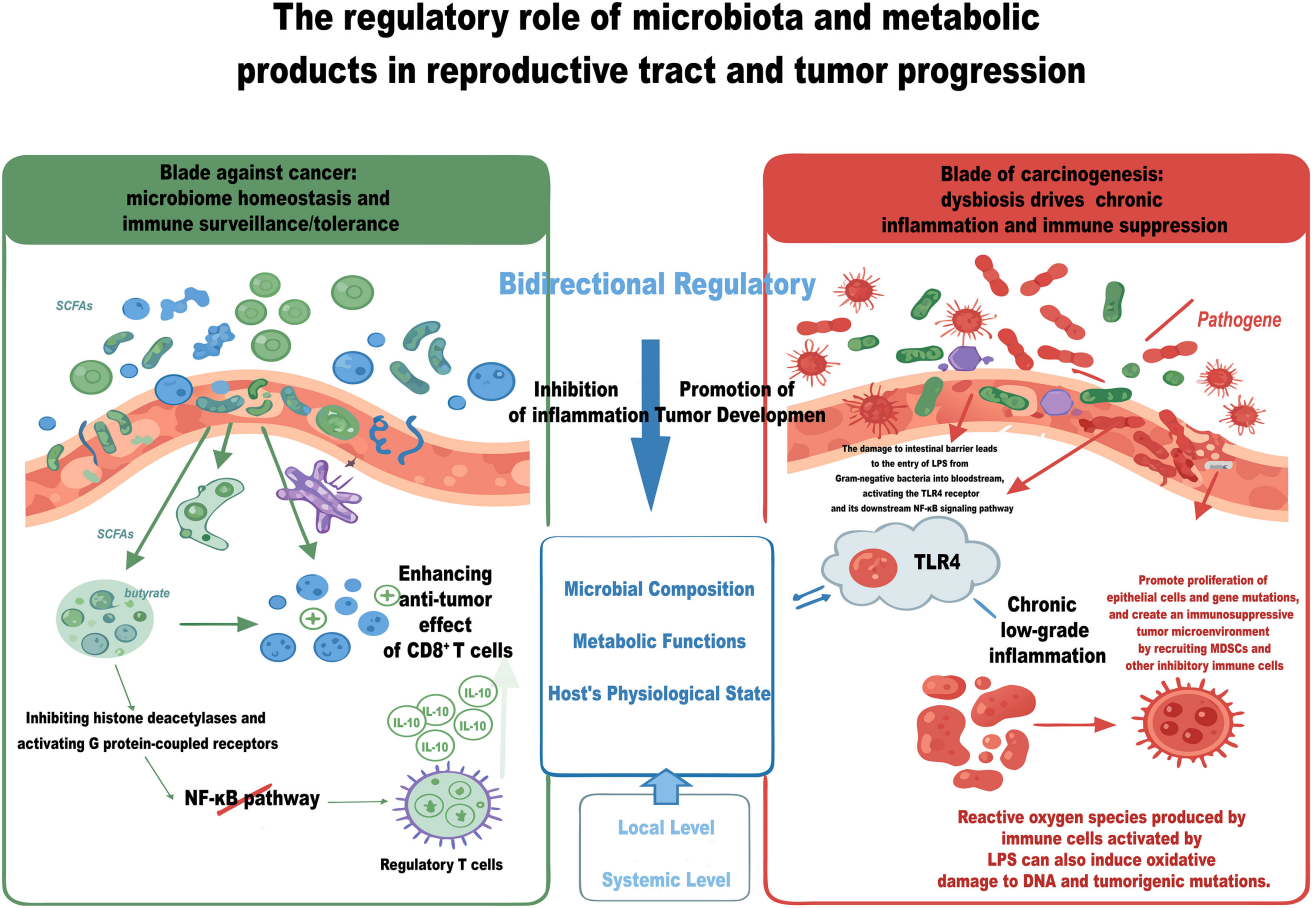
The dual role of microbiota in cancer biology. The microbiota can either sustain immune homeostasis or promote tumorigenesis through distinct mechanisms. For anti-tumor activity, short-chain fatty acids (SCFAs) like butyrate inhibit histone deacetylases and activate G-protein-coupled receptors (GPCRs), leading to suppression of the NF-κB pathway. This promotes IL-10 production by regulatory T cells, mitigating inflammation, and enhances CD8^+^ T cell cytotoxicity and memory through epigenetic reprogramming, thereby bolstering immune surveillance. In contrast, dysbiosis fosters cancer progression. Lipopolysaccharides (LPS) from Gram-negative bacteria breach a compromised intestinal barrier and activate the TLR4/NF-κB signaling pathway, inciting chronic low-grade inflammation. This environment promotes epithelial cell proliferation, genetic mutations, and the establishment of an immunosuppressive tumor microenvironment with myeloid-derived suppressor cells (MDSCs). The balance of these microbial activities is therefore a critical determinant in cancer progression.

The gut microbiota plays a crucial role in the regulation of immune cell functions through its metabolic products. Microbial metabolites are instrumental in determining the fate of innate immune cells; they can modulate the polarization of macrophages and facilitate the differentiation into the M1 phenotype, characterized by its anti-tumor activity. Furthermore, these metabolites can enhance the cytotoxic capabilities of natural killer (NK) cells and promote the maturation of dendritic cells (DCs), thereby improving their antigen-presenting efficacy and establishing a foundation for the initiation of adaptive immune responses (64). In terms of adaptive immunity, this regulatory process exhibits a high degree of context dependence: SCFAs are capable of both promoting the differentiation of Tregs to maintain immune tolerance and enhancing the functionality of effector T cells within TME through specific mechanisms (43, 45). This underscores the ability of the microbiota to achieve precise immune modulation based on microenvironmental signals (**Figure 3**).

#### 3.1.2 Anti-cancer blade: microbiome-mediated immunosurveillance

In a state of eubiosis, the microbiota functions as a critical “endogenous adjuvant,” mediating a state of heightened immunological alertness that is essential for the detection and elimination of nascent malignant cells. This tumor-suppressive capacity is orchestrated through advanced immunological mechanisms that transcend simple immune activation. Effective tumor eradication necessitates CD8^+^ T cells with high metabolic adaptability. Commensal-derived metabolites, particularly butyrate, have been shown to enhance mitochondrial oxidative phosphorylation in CD8^+^ cytotoxic T lymphocytes (CTLs). This metabolic reprogramming mitigates premature exhaustion and preserves a “stem-like” progenitor phenotype (TCF1^+^ CD8^+^), a prerequisite for sustained anti-tumor cytotoxicity and responsiveness to immune checkpoint blockade (45, 65, 66). The microbiota sets the activation threshold for the innate immune system. Tonic commensal signals engage the cGAS-STING pathway to induce basal Type I interferon signaling, which “licenses” NK cells and macrophages. This ensures innate effectors remain poised to rapidly eliminate major histocompatibility complex (MHC)-I-deficient tumor variants that typically escape T-cell recognition (67). Specific commensal bacteria express surface antigens sharing sequence homology with tumor-associated antigens (TAAs). Through molecular mimicry, these microbial antigens prime cross-reactive CD8^+^ T cells within gut-associated lymphoid tissue (GALT). These educationally primed T cells subsequently traffic to distal reproductive tissues to exert specific cytotoxicity, functioning effectively as an autologous cancer vaccine (68). Finally, a healthy microbiome facilitates the neogenesis of tertiary lymphoid structures (TLS) within the reproductive tract mucosa. Mechanistically, commensal-derived signals stimulate stromal cells and DCs to secrete lymphoid chemokines, particularly CXCL13, which recruits CXCR5^+^ B cells and T follicular helper (Tfh) cells to the tumor site. Within these organized ectopic lymphoid aggregates, germinal center-like reactions occur, facilitating *in situ* B-cell somatic hypermutation and the generation of plasma cells producing tumor-specific antibodies. By establishing these local hubs for antigen presentation and lymphocyte maturation, the microbiota transforms the TME from “cold” to “hot”, a feature strongly correlated with favorable prognosis and enhanced responsiveness to immunotherapy in gynecological malignancies (64, 69).

#### 3.1.3 Pro-cancer edge: microbiota dysbiosis drives chronic inflammation and immunosuppression

In stark contrast to physiological immune tolerance, which actively maintains host–microbiota symbiosis under homeostatic conditions, dysbiosis drives a pathological immunosuppressive state that facilitates tumor immune escape. This breakdown of gut microbiota homeostasis unleashes its pro-inflammatory and carcinogenic potential primarily through two pivotal mechanisms. Firstly, LPS serves not only as a crucial structural element maintaining the integrity of the Gram-negative bacterial outer membrane but also as the most distinctive and biologically active microbial molecule detectable within the host circulatory system. The pathogenesis of systemic inflammation in this context is inextricably linked to the ‘LPS/TLR4 axis’. The process initiates with the compromise of intestinal mucosal integrity, often referred to as ‘leaky gut,’ which permits the aberrant translocation of gut-derived LPS from the intestinal lumen into the vascular system. Once in circulation, LPS is recognized by the innate immune system with high specificity. It acts as a canonical ligand for the TLR4, a transmembrane receptor widely expressed on innate immune cells like macrophages and dendritic cells. The ligation of TLR4 mobilizes the canonical NF-κB signaling pathway, a master regulator of inflammation. The subsequent activation of NF-κB promotes the transcription of a vast array of inflammatory mediators, thereby shifting the host’s physiological state from homeostasis to one of chronic, low-grade systemic inflammation—a condition increasingly recognized as a foundational driver of metabolic disorders (45, 70–75). Crucially, this sustained inflammatory milieu acts as a potent driver of carcinogenesis. By inducing oxidative stress and accumulating reactive oxygen species (ROS), it promotes genomic instability and aberrant epithelial proliferation. Concurrently, the release of specific chemokines within this microenvironment orchestrates the infiltration of immunosuppressive populations—notably MDSCs and Tregs—thereby establishing a permissive TME that facilitates immune evasion (76). Researches have shown that immune cells activated by LPS produce reactive oxygen species, and the oxidative DNA damage caused by this may induce tumorigenic mutations (77–79). Secondly, dysbiosis facilitates the synergistic activation of direct carcinogenic cascades, extending its pathological impact far beyond the mere induction of an inflammatory milieu. This causal relationship is substantiated by pivotal fecal microbiota transplantation (FMT) experiments utilizing gnotobiotic murine models. Specifically, the inoculation of germ-free mice with fecal samples from colorectal cancer patients effectively recapitulates the tumorigenic phenotype observed in donors. This process is characterized by aberrant epithelial hyperplasia, profound neovascularization, and the constitutive activation of canonical oncogenic pathways, most notably the Wnt/β-catenin signaling axis. Concomitantly, the induction of a robust Th1/Th17-mediated inflammatory response highlights a critical pathological synergy: microbial communities not only drive genetic instability but also orchestrate immune dysregulation, thereby collectively propelling the neoplastic trajectory (80–83).

#### 3.1.4 The bridge of clinical translation: microbiota modulation of cancer immunotherapy

The profound biological impact of the microbiota is most clinically consequential in the realm of cancer immunotherapy, where it has emerged as a key determinant of therapeutic heterogeneity. Extensive multi-cohort studies have revealed that the gut microbiome composition distinctly stratifies patients into ‘responders’ and ‘non-responders’ to immune checkpoint blockade (ICB). Specifically, a high baseline abundance of immunomodulatory commensals—most notably *Faecalibacterium prausnitzii* and *Akkermansia muciniphila*—is strongly positively correlated with favorable clinical outcomes following programmed cell death protein 1(PD-1)/programmed death-ligand 1(PD-L1) inhibition. Moving beyond mere correlation, FMT experiments have provided elegant causal evidence: transferring stool from responding patients into germ-free mice confers distinct antitumor immunity and restores sensitivity to ICB therapy. Mechanistically, these beneficial microbes are hypothesized to potentiate the therapeutic efficacy by secreting bioactive metabolites and engaging pattern recognition receptors. This interaction primes dendritic cells and facilitates the recruitment of cytotoxic CD8^+^ T cells into the tumor microenvironment, thereby overcoming resistance and amplifying the host’s anti-tumor immune response (64, 84).

Mechanistically, this therapeutic synergy is underpinned by the microbiota’s capacity to augment the antigen-presenting machinery of DCs (85). By promoting DCs maturation and the secretion of pro-inflammatory cytokines (e.g., IL-12), these commensals orchestrate the chemokine-driven recruitment (via CXCL9/CXCL10) and subsequent activation of cytotoxic CD8^+^ T lymphocytes within the tumor bed (84, 86). This cascade effectively reprograms the local immune landscape, converting an immunosuppressive, ‘cold’ tumor microenvironment into an inflamed, anti-tumor phenotype (64, 87). This finding reveals the significant translational potential of regulating the microbiota through strategies such as probiotics, prebiotics, or FMT to improve the efficacy of immunotherapy.

### 3.2 Impact of microbial metabolites on genomic stability

Specific members of the symbiotic microbiota can synthesize secondary metabolites with genotoxic potential. These metabolites can directly induce DNA damage in host cells, thus playing a key initiating role in tumor development (59, 60, 88). Researches have illuminated the role of certain gut bacteria, notably strains of *Escherichia coli* harboring the *pks* gene island, in the synthesis of genotoxins such as colibactin. These metabolites possess the capacity to directly induce double-strand breaks in the DNA of host cells, simultaneously disrupting normal DNA repair processes. This interference culminates in a substantial increase in genomic instability and the accumulation of mutations that are carcinogenic in nature. Such direct insults to genetic material trigger an intricate DNA damage response (DDR) network within the affected cells (88–90). However, when the burden of genotoxic stress overwhelms the capacity of intrinsic DNA repair machinery, or when the fidelity of these reparative pathways is compromised, the inevitable consequence is a progressive escalation of genomic instability. As a cardinal hallmark of cancer, this genomic volatility fosters a permissive mutational landscape that facilitates the acquisition and clonal expansion of oncogenic driver mutations. This process is particularly perilous within high-turnover tissues such as the colorectal epithelium, where rapid cell cycling amplifies the replication of errors, thereby precipitously heightening the trajectory toward malignant transformation (90, 91).

### 3.3 The regulatory role of microbiota in hormone-driven tumors

Microbiota-driven fluctuations in estrogen bioavailability fundamentally recalibrate immune cell polarization within the TME, thereby serving as a decisive factor in tipping the balance between anti-tumor immunosurveillance and immune evasion. In the context of hormone-dependent malignancies, the microbiota functions not merely as a bystander but as a dynamic ‘microbial endocrine organ.’ By regulating host hormone metabolism through the ‘estrobolome’—specifically via enzymatic deconjugation mechanisms—these bacterial communities orchestrate a complex endocrine-immune axis. This regulatory capacity profoundly influences the pathophysiological landscape, dictating whether the local immune milieu remains hostile to tumorigenesis or transitions into a permissive, immunosuppressive niche (33, 35, 92).

#### 3.3.1 Microbial regulation of estrogen levels: core of the enterohepatic circulation

The intestinal microbiota functions as a pivotal regulator of estrogen homeostasis via the enterohepatic circulation, a process governed largely by bacterial β-glucuronidase activity. Following hepatic Phase II metabolism, estrogens are conjugated with glucuronic acid to form hydrophilic, inactive metabolites intended for biliary excretion. However, upon reaching the distal intestine, this elimination pathway is frequently intercepted by the ‘estrobolome’—specifically, bacterial taxa such as *Clostridium* and *Bacteroides* that express high levels of β-glucuronidase. These enzymes catalyze the hydrolysis of the glycosidic bond within conjugated estrogens, effectively deconjugating them to liberate biologically active aglycones. Consequently, these reactivated estrogens are salvaged via reabsorption across the intestinal mucosa, entering the portal circulation to substantially augment the systemic hormonal burden and bioavailability (33–36, 93). This intricate process, governed by specialized microbial genes—collectively termed the estrogen metagenome or estrobolome—represents a pivotal mechanism in maintaining the host’s estrogen homeostasis.

#### 3.3.2 Tumor growth stimulation mediated by dysbiosis

Gut dysbiosis represents a pathological deviation from homeostasis that fundamentally disrupts hormonal metabolism. As demonstrated by Fuhrman et al., this state is clinically characterized by a reduction in microbial αdiversity and a concomitant shift in metabolic function, showing a strong correlation with elevated systemic estrogen levels in postmenopausal women (94). Specifically, the pathological enrichment of taxa possessing high β-glucuronidase activity acts as a catalytic driver for hormonal dysregulation. According to structural biology studies by Ervin et al., these bacterial enzymes exhibit specific loop structures that efficiently bind and hydrolyze conjugated estrogens (36). This aberrant surge in enzymatic activity amplifies the enterohepatic salvage of free estrogens, effectively reversing hepatic clearance and establishing a state of persistent systemic hyperestrogenemia. Consequently, hormone-sensitive distal tissues are chronically subjected to supraphysiological estrogen concentrations. This persistent ligand availability drives the constitutive activation of ERs, initiating a pathogenic signaling cascade. In the context of gynecological malignancies, such as endometrial and ovarian cancers, this “hormonal forcing” fuels downstream mitogenic pathways (e.g., PI3K/Akt and MAPK/ERK) and upregulates pro-proliferative genes like *Cyclin D1* and *MYC*. As elucidated by Walther-António et al., such microbiome-driven hormonal alterations are not merely systemic but are intrinsically linked to the etiology of endometrial hyperplasia and carcinogenesis (95). This creates a permissive microenvironment defined by unchecked cellular proliferation and resistance to apoptosis, providing fertile ground for malignant initiation and clonal expansion (33, 35, 36, 94, 95). Therefore, individuals exhibiting a specific “high-risk” estrobolome signature—characterized by an expanded capacity for estrogen reactivation—face a significantly increased susceptibility to hormone-dependent malignancies. This underscores the critical role of the gut microbiome as an extrinsic endocrine regulator that dictates the host’s cumulative exposure to bioactive estrogens. In summary, the microbiome, alongside its metabolic repertoire, constitutes a complex, multifaceted regulatory nexus that fundamentally orchestrates the pathophysiology of the reproductive tract.

## 4 Evidence and clinical correlation in gynecological cancers

### 4.1 Cervical cancer

Cervical cancer, recognized as the fourth most prevalent malignancy among women globally, represents a formidable public health concern (3, 96). According to recent statistics, there were approximately 660,000 newly diagnosed cases of cervical cancer and nearly 350,000 associated deaths worldwide in 2022 (3). The incidence and mortality rates of this disease are particularly pronounced in developing nations, being 1.7 and 2.4 times higher than those in developed countries, respectively (3). Despite the integration of various modes of treatment—including surgical interventions, radiation therapy, chemotherapy, and immune checkpoint inhibitors—the prognosis for patients with locally advanced cervical cancer remains disheartening, as a significant proportion continues to face the risk of recurrence and mortality (3, 6, 96). Currently, clinical practice is heavily reliant on the International Federation of Gynecology and Obstetrics (FIGO) staging system to direct treatment strategies and evaluate prognostic outcomes. However, these staging systems fundamentally focus on the anatomical progression of the tumor and inadequately encompass the intrinsic biological heterogeneity inherent in the disease (97, 98). At the etiological level, persistent high-risk HPV infection is a necessary precursor for the development of cervical cancer, with detectable viral DNA present in over 99% of cervical cancer specimens. High-risk HPV induces malignant transformation of host cells by disrupting critical tumor suppressor pathways through its oncogenic proteins, E6 and E7 (99). Yet, a perplexing scientific paradox persists: despite the extraordinarily high lifetime infection rate of high-risk HPV among sexually active women worldwide, approximately 90% of these infections are transient and can be effectively resolved by the host immune system within 8 to 24 months. Only about 10-15% of high-risk HPV infections progress to persistent states, which are crucial in the development of cervical intraepithelial neoplasia (CIN) and invasive cancer. This phenomenon raises a pivotal scientific inquiry: aside from high-risk HPV acting as the initiating agent, what host factors create a conducive environment for its persistence and subsequent carcinogenesis (100, 101). In recent years, research efforts have expanded beyond the local cervical microenvironment to encompass the broader gut–vaginal axis, a bidirectional communication system that fundamentally regulates reproductive health. To clarify the distinct roles within this crosstalk: the healthy gut microbiota serves as a diverse reservoir that maintains systemic immune homeostasis, whereas the healthy cervicovaginal microbiome is uniquely characterized by low diversity and the dominance of Lactobacillus species, which maintain a protective acidic pH. However, an emerging body of evidence highlights that disruption in this axis—often initiating in the gut—can precipitate cervicovaginal dysbiosis. Unlike the gut, where high diversity is beneficial, a shift towards high microbial diversity in the cervix (loss of *Lactobacillus* dominance) represents a pathological state. This local dysbiosis, potentially fueled by systemic signals from the gut, plays a critical and synergistic role in the cascade of events leading to persistent high-risk HPV infection. Thus, this dysbiosis forms a vital ‘soil,’ conditioned by both local and systemic microbial alterations, that enables the ‘roots’ of HPV infection to take hold and progress toward cervical carcinogenesis (101–104). This groundbreaking discovery has profound implications for our understanding of cervical cancer and compels us to reevaluate the significance of microbiota in the etiology and development of such diseases.

#### 4.1.1 The neglected soil: vaginal microecological imbalance as a permissive niche for HPV

The cervicovaginal microenvironment functions as a primary gatekeeper against viral acquisition. In a state of optimal health—often classified as CST I—the niche is dominated by *Lactobacillus crispatus*. Current mechanistic evidence suggests that *L. crispatus* confers colonization resistance not merely through acidification, but via the stereospecific production of D-lactic acid. Unlike its L-isomer, D-lactic acid has been shown in preclinical models to downregulate extracellular matrix metalloproteinase inducer (EMMPRIN) and inhibit matrix metalloproteinase-8 (MMP-8). This stereospecific regulation reinforces the viscosity of cervicovaginal mucus and preserves the integrity of epithelial tight junctions, creating a robust physicochemical “firewall” that physically impedes viral access to basal keratinocytes (101, 103–105). In contrast, vaginal dysbiosis (CST IV), characterized by a high-diversity polymicrobial biofilm of obligate anaerobes, represents a critical ecological shift associated with viral pathogenesis. It is imperative to rigorously distinguish epidemiological correlation from etiological causation: while extensive longitudinal human cohorts have established a robust statistical link between CST IV and increased HPV acquisition, definitively proving that dysbiosis functions as a primary driver rather than a consequence of infection remains challenging. However, *in vitro* investigations provide a biologically plausible rationale for this association, proposing a mechanism of Enzymatic Barrier Breach. Dysbiotic pathobionts—notably *Gardnerella vaginalis*—secrete high concentrations of sialidases and mucinases. These enzymes hydrolyze the terminal sialic acid residues on host mucins, dismantling the gel-forming capacity of the mucus. This enzymatic degradation effectively “unlocks” the physicochemical firewall, physically facilitating the penetration of HPV virions to their target receptors (98–101, 103, 104, 106–109). Beyond physical barrier disruption, dysbiosis triggers a local inflammatory response characterized by elevated IL-1β, IL-6, and TNF-α (109). Paradoxically, this non-specific inflammation does not clear the virus; instead, it creates a “permissive microenvironment” by recruiting activated immune cells that serve as targets for coinfections or inducing ROS. These ROS contribute to host DNA damage, thereby synergizing with HPV oncogenes (E6/E7) to promote genomic instability (108, 110). Recent advancements have underscored that “*Lactobacillus*-dominance” is not universally protective; the immunological outcome is strictly dictated by species-specific functional profiles. *L. crispatus* relies on the unique D-lactic acid-mediated suppression of EMMPRIN/MMP-8 to maintain barrier integrity (105). In stark contrast, *L. iners*-dominance (CST III) represents an ecologically ambiguous state. Unlike *L. crispatus*, *L. iners* primarily produces the L-lactic acid isomer, which is ineffective at downregulating EMMPRIN, resulting in a comparatively “leaky” mucosal barrier. Furthermore, *L. iners* expresses inerolysin, a cholesterol-dependent cytolysin (CDC). This pore-forming toxin may compromise epithelial membrane integrity and induce sub-clinical inflammation. Consequently, *L. iners* is epidemiologically associated with significantly higher rates of HPV recurrence, highlighting it as a marker of mucosal vulnerability rather than stability (111–113). In the specific niche of the local cervical TME, the enrichment of specific anaerobic genera—notably *Sneathia*, *Fusobacterium*, and *Porphyromonas*—constitutes a consistent microbial biosignature of malignancy. However, interpreting this shift from a healthy *Lactobacillus*-dominant state presents a critical epistemological challenge. Current mechanistic models propose a bidirectional relationship: these taxa may function as active ‘drivers’ of tumorigenesis by generating immunomodulatory oncometabolites—such as succinate (which stabilizes HIF-1α) and polyamines—or by engaging immune checkpoints (e.g., *Fusobacterium*-TIGIT interaction). Conversely, they may exist merely as opportunistic ‘passengers’ that exploit the hypoxic, necrotic niche created by the tumor (‘tumorotropic colonization’). Given this etiological complexity, therapeutic strategies aiming for local ecological restoration—specifically vaginal microbiota transplantation (VMT)—are currently classified as investigational approaches. While supported by strong preclinical rationale, rigorous randomized controlled trials (RCTs) are urgently required to determine whether re-establishing physiological *L. crispatus* dominance translates into tangible oncological benefits (108, 110, 114).

#### 4.1.2 Accelerators of carcinogenesis: role of vaginal microbiome dysbiosis in cancer development and progression

Vaginal microbiome dysbiosis is not merely a background factor for the occurrence of cervical cancer; rather, it promotes the development of cervical cancer through various interrelated biological mechanisms (101, 103, 104).

The dysregulation of cervical microecology is often marked by a diminishment in protective *lactobacilli* and an aberrant proliferation of various anaerobic bacteria. This imbalance serves as a primary catalyst for chronic local inflammation (115, 116). The pathogen-associated molecular patterns (PAMPs) released by these anaerobic organisms effectively engage pattern recognition receptors (PRRs) present on the surfaces of epithelial and immune cells, initiating a cascade of downstream inflammatory signaling pathways, notably including NF-κB (117). This activation culminates in the persistent release of a hierarchically organized cytokine network: pro-inflammatory cytokines, such as IL-1β, TNF-α, IL-6, drive chronic inflammation and tissue damage, while chemotactic cytokines, such as interferon-inducible protein-10 (IP-10/CXCL10) and macrophage inflammatory protein-1α (MIP-1α/CCL3), actively recruit leukocytes to the lesion site, perpetuating the inflammatory loop (115–117). Together, these cytokine classes cooperate to generate a paradoxical microenvironment characterized by simultaneous inflammation and immune tolerance. This chronic inflammatory milieu not only invites substantial infiltration of immune cells but also enables the inflammatory mediators to foster abnormal cell proliferation, impede apoptosis, and heighten genomic instability. Consequently, this creates a conducive environment for the carcinogenesis of cells infected by HPV.

The progression from persistent viral infection to invasive malignancy necessitates the active subversion of local immune surveillance, a process facilitated by dysbiosis through the construction of a multi-layered immunosuppressive network. A pivotal evasion mechanism involves the direct engagement of inhibitory receptors on cytotoxic cells; specifically, *Fusobacterium nucleatum* actively subverts antitumor immunity by expressing the surface protein Fap2, which binds to the T cell immunoreceptor with Ig and ITIM domains (TIGIT) on NK and T cells. Unlike physiological regulatory mechanisms, this bacterial engagement triggers an aberrant inhibitory signal acting as a potent ‘off-switch.’ By directly attenuating cytotoxic granule release, *F. nucleatumcreates* an immunosuppressive niche that functionally paralyzes the host’s ability to eliminate HPV-infected cells (118, 119). Concurrently, the dysbiotic microenvironment orchestrates a detrimental shift in the local cytokine milieu, where the enhanced secretion of immunosuppressive cytokines—notably transforming growth factor-beta 1 (TGF-β1) and IL-10—promotes the differentiation of Tregs while suppressing antiviral effector T cells. This cytokine profile drives a “functional skewing” from a protective Th1 response (required for viral clearance) to a permissive Th2/Treg-dominant phenotype (120, 121). Furthermore, exposure to dysbiotic signals can induce the upregulation of PD-L1 on tumor cells, delivering co-inhibitory signals that halt T-cell activation. In summary, dysbiosis cultivates a paradoxical “inflammatory-suppressive” microenvironment where chronic NF-κB-driven inflammation fuels mutagenesis, while the simultaneous activation of immune checkpoints and suppressive cytokines safeguards these aberrant cells from elimination, creating a sanctuary for HPV persistence and malignant progression (120–122). In summary, dysbiosis engenders not merely a singular inflammatory or suppressive response but rather cultivates a paradoxical yet harmonious pro-cancer microenvironment. Within this setting, chronic inflammation perpetually drives the malignant transformation of cells, while the concurrently robust immunosuppressive mechanisms safeguard these aberrant cells from detection and elimination by the immune system. This synergistic state of heightened inflammation and immunosuppression creates a sanctuary for the persistent infection of HPV and the advancement of precancerous lesions, forming the fundamental pathophysiological basis for the erosion of local immune surveillance.

#### 4.1.3 Alteration of local metabolic profiles

Distinct from the systemic immunomodulatory influence of the gut microbiota, the local cervicovaginal microbiota exerts a direct and specific impact on the chemical composition of the reproductive tract microenvironment. In a healthy state, a resident *Lactobacillus*-dominated community maintains homeostasis through the production of beneficial metabolites, such as anti-inflammatory nucleotides (108, 110). However, the transition to precancerous lesions is characterized by a shift in this local ecosystem, where dysregulation of amino acid and nucleotide metabolism mirrors the interplay between a dysbiotic microbiome and host cellular hyperproliferation (110, 123). In the oncogenic stage, the intratumoral and vaginal microenvironments undergo significant lipid metabolic reprogramming (124, 125). This metabolic alteration enables cancer cells—synergistically supported by the altered local microbiome—to enhance *de novo* fatty acid synthesis, thereby securing the membrane components and signaling molecules required for rapid proliferation. Furthermore, local dysbiosis may sustain this oncogenic phenotype by creating a nutrient-rich biochemical niche that favors tumor cell survival (126, 127). Collectively, cervical carcinogenesis is driven by a complex, multifaceted cascade involving HPV infection, host cellular responses, and the vaginal microenvironment. As our comprehension of these dynamics deepens, clinical paradigms are evolving toward more precise, proactive methodologies. Elucidating the tripartite crosstalk among specific bacterial taxa, viral oncoproteins, and host signaling pathways is essential for uncovering the synergistic mechanisms of pathogenesis. In this context, next-generation risk assessment models are emerging that transcend traditional HPV genotyping. By integrating vaginal microbiome signatures with immune and metabolic biomarkers, these advanced systems aim to achieve multidimensional risk stratification. Such an integrated approach not only enhances the sensitivity of early screening but also informs individualized therapeutic strategies. Crucially, this conceptual evolution underpins the development of novel microbiome-based interventions. Modulating the cervicovaginal microenvironment—through probiotics, postbiotics, or targeted pharmacological agents to restore *Lactobacillus* dominance—offers a promising avenue to arrest the progression of precancerous lesions. This transition from passive screening to proactive microbiome-mediated interception represents a paradigm shift in the management of cervical cancer, offering a robust strategy for disease mitigation.

### 4.2 Endometrial cancer

The global epidemiological landscape of endometrial cancer is undergoing a profound transformation. According to the 2022 global cancer statistics, approximately 420,000 new cases were diagnosed that year. Over the past decade, the age-standardized incidence rate has surged by around 25%, a trajectory that ranks among the highest for gynecological malignancies (3). This notable increase is closely associated with several sociodemographic factors: the advancing process of global population aging, a persistent rise in the prevalence of metabolic syndrome driven by dietary and lifestyle changes, and evolving patterns in female reproductive behaviors. Particularly noteworthy is the recent emergence of a bimodal age distribution, which defies traditional paradigms by indicating a simultaneous escalation in incidence risk for both women of childbearing age and postmenopausal women (8, 128). Recent advancements in research have illuminated that the etiology of endometrial cancer cannot be solely attributed to conventional endocrine factors. Instead, it is now recognized as the result of a positive feedback loop governed by the microbiota-estrogen-inflammation axis. Dysbiosis of the microbiota plays a crucial role in initiating and exacerbating this feedback loop. In summary, the evolving understanding of endometrial cancer’s etiology and increasing incidence underscores the pressing need for ongoing research and targeted interventions to address the significant public health concern (129–131).

#### 4.2.1 Dysbiosis of microbiota and systemic changes

Dysbiosis exerts a profound influence on systemic pathophysiological alterations associated with the development of endometrial cancer through two primary mechanisms (35, 93). First, at the level of estrogen metabolism, specific bacterial taxa within the gut microbiota, notably *Bacteroides* and *Clostridium*, produce β-glucuronidase. This enzyme specifically hydrolyzes estrogen-glucuronide conjugates synthesized by hepatic enzymes UGT1A1 and UGT2B7. Such enzymatic action reactivates dormant estrogen, originally earmarked for biliary excretion, converting it back into its biologically active free form. These reactivated estrogen molecules subsequently enter the portal venous system via passive diffusion across the intestinal epithelial barrier, thereby completing the enterohepatic circulation and resulting in a significant augmentation of bioactive estrogen levels within the circulatory system (93, 132–134). Crucially, this unopposed hyperestrogenemia exerts a dual oncogenic effect: it directly stimulates abnormal endometrial cell proliferation while simultaneously signaling through immune receptors to dampen anti-tumor surveillance (135–137). Dysbiosis, alongside associated metabolic irregularities, can initiate systemic chronic inflammatory responses. In pathological states such as obesity, the integrity of the gut barrier is compromised, leading to augmented intestinal permeability. This compromised state permits substantial amounts of LPS—essential constituents of the cell walls of Gram-negative bacteria—to traverse into the circulatory system, consequently resulting in elevated plasma concentrations of LPS (70, 138, 139). These endotoxins bind to TLR4 and its downstream NF-κB signaling pathway, activating the mononuclear-macrophage system and leading to the release of a large number of pro-inflammatory cytokines, thereby establishing a state of systemic chronic low-grade inflammation. This persistent inflammatory microenvironment not only increases genomic instability by producing ROS but also promotes cell proliferation and angiogenesis, creating conditions conducive to tumor initiation and progression (129, 140–142).

#### 4.2.2 Vaginal/uterine microbiome dysbiosis and local microenvironment disruption

In the pathogenesis of endometrial cancer, local dysbiosis of the vaginal and uterine microbiome constitutes a critical promoting factor. This stage is characterized by a structural imbalance in the reproductive tract microecology: protective *Lactobacillus* species are significantly depleted, while strictly anaerobic pathogenic bacteria such as *Atopobium vaginae*, *Porphyromonas somerae*, and *Dialister microerophilus* exhibit several-fold abnormal enrichment. These changes in microbial composition directly led to the collapse of the local defense system— the decrease in lactic acid concentration produced by *Lactobacillus* raises the vaginal pH from a physiological range of 3.8-4.2 to a pathological range of 4.5-6.0, disrupting the acidic environment that inhibits the growth of pathogenic bacteria (33, 62, 92, 143).Simultaneously, the expression levels of the antimicrobial peptide LL-37 and the concentration of IgA on the mucosal surface exhibited a marked reduction of approximately 60% and 45%, respectively. This substantial decline significantly undermined the local physical and immune barrier functions. In light of such a comprehensive weakening of defensive mechanisms, opportunistic pathogens within the vagina were afforded the opportunity for retrograde ascension, successfully breaching the cervical barrier and colonizing the uterine cavity. This process created the requisite microenvironmental conditions that could facilitate subsequent carcinogenic processes. A recent comprehensive narrative review by Aquino et al. has provided critical evidence reinforcing this pathological transition. By systematically comparing the microbiota status between patients affected by endometrial cancer and healthy controls, the authors highlighted that the deviation from eubiosis is distinct and quantifiable. They emphasized that affected patients exhibit a specific “microbial signature” characterized not only by the depletion of protective *Lactobacillus* species but also by a correlating shift in vaginal pH and inflammatory markers compared to healthy individuals. This “affected versus healthy” dichotomy suggests that specific dysbiotic patterns—such as the overgrowth of *Atopobium* and *Porphyromonas*—are not merely concurrent features but may serve as early indicators of the carcinogenic cascade. Integrating these findings confirms that monitoring the microbiota status offers a novel lens for distinguishing high-risk populations from healthy cohorts (144). Current researches have unequivocally established the pivotal role of the microbiota in the etiology of endometrial cancer. The microbiota not only influences cellular proliferation through the regulation of estrogen metabolism but also fosters genomic instability via the activation of inflammatory pathways. Moreover, it modifies the characteristics of the TME through its metabolic byproducts (54, 145, 146). These findings provide a novel biological foundation for elucidating health disparities across different racial groups and underscore the potential for developing innovative prevention and treatment strategies centered on microenvironmental regulation.

#### 4.2.3 Microbiota drives malignant transformation

Pathogenic bacteria that colonize the uterine cavity play a pivotal role in the malignant transformation of endometrial cells through a complex array of molecular mechanisms. At the inflammatory activation level, *Atopobium vaginae* interacts with TLR 2 via its surface lipoproteins, initiating MyD88-dependent signaling pathways that facilitate the nuclear translocation of the NF-κB transcription factor. This process triggers a cascade of pro-inflammatory cytokines, including IL-6, IL-8, and TNF-α, thereby establishing a persistently inflammatory microenvironment (145, 147). In terms of epigenetic regulation, butyrate produced through the metabolism of *Porphyromonas somerae* exerts an inhibitory effect on HDACs. This results in conformational changes in chromatin that impact the transcriptional expression of genes associated with DNA damage repair. Furthermore, concerning genomic stability, ROS generated during microbial metabolism can inflict oxidative damage on DNA, significantly heightening the mutation burden of critical tumor suppressor genes such as *PTEN* (132, 148). Additionally, metalloproteases and other proteolytic enzymes secreted by pathogenic bacteria have the capacity to specifically degrade tight junction proteins, such as occludin and ZO-1. This degradation disrupts the integrity of the endometrial epithelial barrier, thereby perpetuating a vicious cycle of infection, inflammation, and tissue damage (149–151). Collectively, these synergistic molecular events establish the fundamental pathological and physiological framework through which microorganisms facilitate the development of endometrial cancer.

In summary, the occurrence and progression of endometrial cancer is a complex pathological and physiological process driven by the interplay of systemic endocrine disorders, local microenvironment disruptions, and dysbiosis of microbiota. The core factor is the sustained high levels of estrogen, which act as a classical driving force by activating estrogen receptor signaling pathways, providing the initial signal for the abnormal proliferation of endometrial cells. At the same time, microbiota dysbiosis originating from the vagina or intestine plays a crucial role as a multiplier and accelerator. The three mechanisms—hormonal drive, inflammatory damage, and direct microbial action—are interwoven, forming a powerful positive feedback loop that collectively promotes the transformation of endometrial tissue from a normal state through hyperplasia and atypical hyperplasia, ultimately accelerating the transition to a malignant phenotype. This new understanding, which spans from the systemic to the local and from the macro to the micro, not only provides a powerful new microbiological tool for the early screening and stratification of high-risk populations for endometrial cancer but also lays a solid theoretical foundation for future disease prevention and treatment through targeted regulation of the microecological environment, such as the application of probiotics.

### 4.3 Ovarian cancer

Ovarian cancer is the deadliest gynecological cancer, and clinical management faces two major challenges. Firstly, the biological characteristics of the disease make early diagnosis difficult; approximately 70% of patients are already diagnosed at stage III or IV, missing the optimal treatment window. Secondly, although most patients can achieve remission with initial surgery combined with platinum-based chemotherapy, up to 80% of late-stage patients will face platinum resistance and recurrence, resulting in stagnation of the five-year survival rate. Despite advancements in immunotherapy for other solid tumors, its effectiveness in ovarian cancer has been poor, reflecting a highly immunosuppressive TME (4, 5, 152, 153). Therefore, reversing chemotherapy resistance and breaking the immunosuppressive microenvironment are fundamental to improving the prognosis of ovarian cancer patients. For a long time, research on ovarian cancer has primarily focused on genetic mutations in tumor cells and host genetic factors. However, these factors fail to fully explain the tumor heterogeneity, the differences in treatment responses, and the formation of the microenvironment. This limitation has prompted scientists to turn the attention to the non-host factors in the tumor ecosystem—the human symbiotic microbiome. The shift in perspective provides a new theoretical framework for understanding the complexity of ovarian cancer.

#### 4.3.1 Dysbiosis and its association with ovarian cancer risk, staging, and prognosis

**Figure 4:**
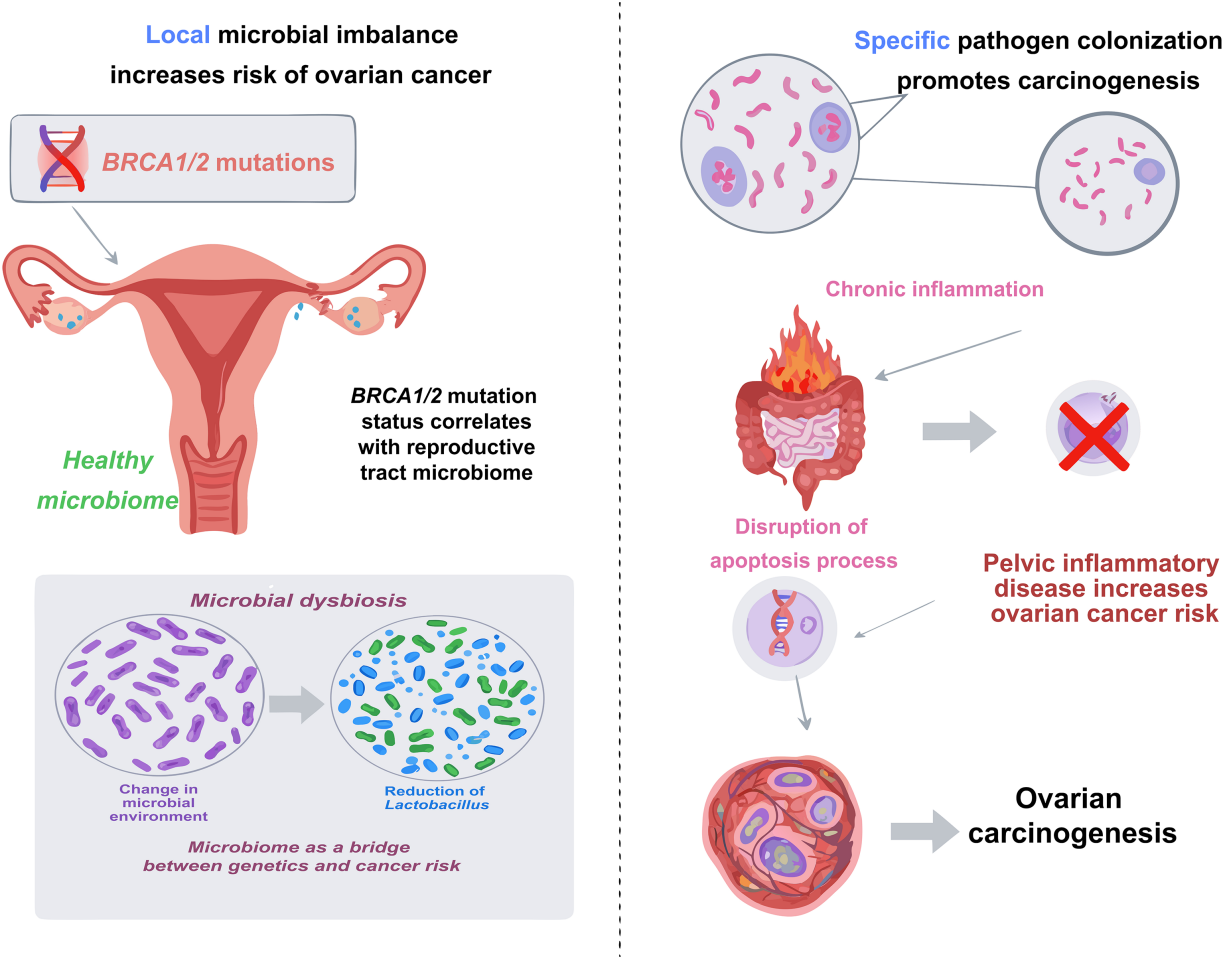
The interplay of host genetics, microbiome, and pathogens in ovarian cancer pathogenesis. Ovarian cancer etiology involves a complex integration of genetic and microbial determinants. Host genetic background, particularly *BRCA1/2* mutation status, correlates with distinct cervicovaginal dysbiosis characterized by the depletion of protective *Lactobacillus*, thereby establishing a pro-tumorigenic microenvironment. Concurrently, specific pathogens exert direct oncogenic effects; notably, *Chlamydia trachomatis* interferes with p53 signaling to abrogate apoptosis, facilitating the survival of cells harboring DNA damage and promoting carcinogenesis.

Recent advancements have shifted the focus from isolated local dysbiosis to the concept of the gut–vaginal microbiome axis, a bidirectional communication network that critically influences ovarian cancer pathogenesis. This crosstalk suggests that the gut acts as an extra-vaginal reservoir for bacterial populations and modulates the vaginal microenvironment through metabolic and immune signaling, thereby impacting cancer susceptibility and progression. Genetic susceptibility, particularly *BRCA1* and *BRCA2* mutations, remains a cornerstone of ovarian cancer risk (4, 5). However, emerging evidence indicates that these genetic factors do not act in isolation but significantly shape the gut–vaginal microbial landscape (154, 155). A key case-control study revealed that *BRCA1* mutation carriers, particularly those under 50, exhibit a distinct “Community State Type O” (CST-O), characterized by a marked depletion of protective *Lactobacillus* species in the cervicovaginal niche. Notably, this loss of local dominance is often mirrored by or linked to gut dysbiosis, where the “estrobolome”—gut bacteria capable of metabolizing estrogens—may alter systemic hormone levels, further disrupting vaginal homeostasis (23) This data suggests that the microbial microenvironment serves as a crucial mediator between genetic predisposition and tumorigenesis (**Figure 4**) (23, 156–158). The gut–vaginal crosstalk implies that dysbiosis in one compartment may trigger or sustain dysbiosis in the other, creating a systemic pro-tumorigenic environment. Consequently, simultaneous profiling of both gut and vaginal microbiomes could offer a more robust strategy for early risk stratification in *BRCA* carriers than analyzing either site alone.

Chronic infections caused by specific pathogens are considered one of the risk factors for ovarian cancer. The mechanisms involved include the induction of chronic inflammation and the disruption of normal cellular apoptosis processes, which can promote carcinogenesis. Several studies have confirmed a significant association between *Chlamydia trachomatis* infection and an increased risk of ovarian cancer. Serological tests have shown that positive antibodies against specific proteins of *Chlamydia trachomatis* (Pgp3) are associated with approximately a two-fold increase in risk of ovarian cancer (159–161). This association is specific, as antibodies against other common pathogens have not shown an association with risk of ovarian cancer (159, 160). The potential carcinogenic mechanism of *Chlamydia trachomatis* may be associated with its capacity to inhibit apoptosis in host cells. Research has indicated that *Chlamydia trachomatis* disrupts the functionality of the tumor suppressor protein p53, thereby obstructing the apoptotic process in infected cells. Under conditions of DNA damage, *p53* is responsible for initiating repair mechanisms or triggering apoptosis to avert the development of cancer cells. By inhibiting the action of *p53*, *Chlamydia trachomatis* fosters an environment conducive to its own replication within host cells, while simultaneously permitting the survival of cells that harbor accumulated DNA damage. The paradigm raises the likelihood of these damaged cells ultimately progressing toward carcinogenesis (162). Chronic inflammation caused by infections, such as pelvic inflammatory disease (PID), is an important risk factor for the increased risk of ovarian cancer associated with *chlamydia* infection (161, 163, 164). In addition to *Chlamydia trachomatis*, other intracellular microorganisms that can cause chronic infections have also been found to be associated with ovarian cancer. Researchers have identified pathogens such as *Brucella, Mycoplasma, and Chlamydia* in ovarian cancer tumor cells (163, 165). A recent systematic review and meta-analysis highlighted evidence demonstrating a significantly higher prevalence of *Chlamydia trachomatis* DNA in ovarian cancer tissues than in benign ovarian tumors (166). While research into the relationship between these pathogens and ovarian cancer is still evolving, current evidence indicates that both the prevention and treatment of chronic infections caused by these pathogens could potentially mitigate the risk of ovarian cancer in certain women (164, 165). Therefore, integrating the detection of specific pathogens with an assessment of the broader gut–vaginal microbial network holds significant promise. This dual approach could not only elucidate the etiology of “infection-associated” ovarian cancers but also lead to novel early diagnostic panels that combine pathogen screening with microbiome signatures (**Figure 4**).

The tumor-associated microbiome emerges as a dynamic driver of oncogenesis rather than a passive bystander, actively dictating the immunological configuration of ovarian cancer. Ovarian cancer typically manifests as a non-immunogenic “cold” tumor, characterized by the exclusion of cytotoxic T cells and the dominance of immunosuppressive myeloid populations. Specific intratumoral pathogens actively enforce this immune-excluded state. Researches have highlighted that *Acinetobacter* species can drive the polarization of tumor-associated macrophages (TAMs) towards an immunosuppressive M2 phenotype. These M2-TAMs secrete TGF-β and IL-10, forming a dense stromal barrier that physically and chemically excludes CD8^+^ T cells from tumor islets, thereby maintaining an inflammatory state conducive to immune evasion (153, 167). Beyond direct immune suppression, intratumoral dysbiosis hijacks developmental signaling pathways to fuel progression. Notably, *Cutibacterium acnes* (formerly *Propionibacterium acnes*) has been identified as a pivotal promoter of malignancy. Mechanistically, inflammatory responses elicited by *C. acnes* trigger the aberrant activation of the Hedgehog (Hh) signaling pathway, elevating the expression of critical proteins including Shh, Gli1, and Gli2. This reactivation of dormant developmental pathways synergizes with chronic inflammation to accelerate cellular proliferation and invasion (167–169). Conversely, modulation of the microbiota offers a pathway to reverse this aggressive phenotype. Favorable commensal signals (e.g., STING agonists) can stimulate DCs to produce Type I Interferons, which upregulate T-cell-attracting chemokines (CXCL9, CXCL10). This establishes a chemokine gradient that facilitates the trafficking of effector T cells into the tumor core, transforming the “cold” immune desert into an immune-inflamed “hot” tumor. This microbial-driven immunological switch is increasingly recognized as a critical determinant of patient prognosis and therapeutic response (67, 170–172).

#### 4.3.2 The impact of microbial communities on the sensitivity and resistance to chemotherapy in ovarian cancer

The microbial communities in the gut and local tumor environment significantly influence the effectiveness of first-line chemotherapy for ovarian cancer through various complex mechanisms. These microbial communities can not only directly induce drug resistance in cancer cells but also indirectly weaken the efficacy of chemotherapy by regulating systemic immunity and inflammatory responses.

LPS, a critical component of the cell wall of *Escherichia coli* (*E. coli*), has been identified as a significant contributor to chemotherapy resistance in cancer. Research indicates that TLR4, which is expressed on the surface of ovarian cancer cells, is capable of recognizing LPS. Upon binding of LPS to TLR4, the downstream MyD88 and NF-κB signaling pathways are activated. This activation triggers tumor cells to secrete a variety of pro-inflammatory cytokines and chemokines, thereby creating a microenvironment that is conducive to tumor growth. Notably, these factors also instigate anti-apoptotic mechanisms, enabling cancer cells to withstand apoptosis induced by chemotherapy agents such as paclitaxel, ultimately leading to resistance to such therapeutic interventions (173). Therefore, a pro-inflammatory microenvironment composed of specific microorganisms can directly weaken the effectiveness of chemotherapy by continuously activating inflammatory signals.

In addition to directly inducing resistance, the microbiome can also influence chemotherapy effectiveness through more macro-level regulation. During chemotherapy, this imbalanced immune state can interfere with the antitumor effects of chemotherapy drugs (174, 175). Clinical studies have found that ovarian cancer patients who are resistant to platinum-based chemotherapy exhibit significant differences in gut microbiota diversity and composition compared to those who are sensitive to platinum. The gut microbiota diversity is markedly reduced in resistant patients (176). The use of broad-spectrum antibiotics during chemotherapy significantly disrupts the gut microbiota, and this phenomenon is importantly associated with poorer treatment responses and survival rates in ovarian cancer patients. This further validates the critical role of a complete gut microbiome in maintaining chemotherapy sensitivity (177, 178).

Epithelial-mesenchymal transition (EMT) is a key biological process through which cancer cells acquire invasive and metastatic abilities as well as chemoresistance (179). Animal model studies have shown that dysbiosis of the gut microbiota may promote the occurrence of EMT. The mechanism behind this phenomenon may be closely related to the damage of the intestinal barrier function. Dysbiosis allows bacterial products, such as LPS, to enter the bloodstream, thereby activating peripheral immune cells, particularly macrophages. The activated macrophages then release a large number of pro-inflammatory factors, such as tumor TNF-α and interleukin-6, further exacerbating the inflammatory response (180). These inflammatory factors in the cycles act on tumor cells, inducing EMT, thereby enhancing their invasive capabilities and leading to resistance to chemotherapy drugs (153, 181).

#### 4.3.3 Potential role of microbiota in immunotherapy for ovarian cancer

The microbiota, whether present in the gut or locally within tumors, is a key factor in regulating host immune responses. Its composition and function profoundly affect the efficacy of anti-tumor therapies, including poly ADP-ribose polymerase (PARP) inhibitors and immune checkpoint inhibitors. PARP inhibitors are a cornerstone therapy for *BRCA*-mutated ovarian cancer. The efficacy is not only reliant on directly killing cancer cells with homologous recombination deficiency (HRD) through the synthetic lethality effect, but also partially depends on eliciting effective anti-tumor immune responses. The microbiome, as an important regulator of systemic and local immunity, can shape the TME, thereby indirectly influencing the effectiveness of PARP inhibitors. Research has shown that the composition of the gut microbiome is associated with the therapeutic effects of PARP inhibitors. In a study involving ovarian cancer patients treated with PARP inhibitors, a higher abundance of bacteria from the genus *Phascolarctobacterium* was significantly associated with longer progression-free survival (PFS) in *BRCA* wild-type patients (182). This indicates that the microbiome may exert an influence on patients’ responses to targeted therapy by modulating the immune system.

Chronic inflammation driven by persistent infections from certain pathogens or dysbiosis typically creates an immunosuppressive TME, thus weakening the efficacy of immunotherapy. Some intratumoral bacteria, have been found to be negatively correlated with the infiltration of M1 pro-inflammatory macrophages and can inhibit the migration of macrophages, which may lead to a diminished antitumor immune response. The chronic inflammatory state maintained by dysbiosis recruits a large number of MDSCs and M2 tumor-associated macrophages, which are key immunosuppressive components in the tumor immune microenvironment. The presence is detrimental to the optimal immune-activating effects of therapies like PARP inhibitors (183, 184). In stark contrast to pathogenic bacteria, certain strains of lactic acid bacteria have demonstrated the remarkable capability to bolster the host’s anti-tumor immune response. Notably, research indicates that *Lactobacillus gallinarum* can impede the differentiation of immune-suppressive Tregs within the TME through the modulation of tryptophan metabolism (185). Simultaneously enhance the functionality of cytotoxic CD8^+^ T cells, thereby significantly improving the efficacy of anti-PD-1 immunotherapy in animal models (84). The significant depletion of *lactobacilli* observed in ovarian cancer patients may indicate a weakening of local immune surveillance, which not only promotes tumor development but may also affect the efficacy of various anticancer therapies, including PARP inhibitors and immune checkpoint inhibitors.

## 5 Innovative strategies for diagnosis and treatment targeting gut microbiota

### 5.1 The microbiome as a biomarker

#### 5.1.1 Cervical cancer: microbiome as a potential sentinel for disease progression

Research has demonstrated a noteworthy alteration in the intestinal microbiota of cervical cancer patients, characterized by a significant increase in the relative abundance of genera such as *Prevotella*, *Porphyromonas*, and *Dialister*. Concurrently, there is an observable decline in the prevalence of genera including *Bacteroides*, *Alistipes*, and various members of the family *Lachnospiraceae*. These findings underscore the promising potential of the microbiome as a valuable tool for monitoring disease progression, offering novel insights into the early diagnosis and efficacy assessment of cervical cancer (186). This dysbiosis may create conditions for cancer cell escape by weakening the immune surveillance function of the host. Predictive models based on these specific microbial characteristics have shown high accuracy in the diagnosis of cervical cancer (187).

#### 5.1.2 Endometrial cancer: interplay of dysbiosis and hormonal metabolism

The occurrence of endometrial cancer is closely related to the host’s metabolism and hormonal environment, with the gut microbiota playing a central role in regulating these two systems. Therefore, an in-depth analysis of the composition of the microbiota not only aids in understanding the mechanisms behind this disease but also highlights its significant potential as a novel, non-invasive biomarker. Research findings clearly indicate that the presence of specific microbial communities is directly associated with the risk of endometrial cancer. For instance, elevated levels of bacteria from the genera *Marvinbryantia*, *Ruminococcaceae UCG014*, and *Dorea* have been detected in European populations, which may suggest a lower risk of the disease and thus can be considered protective biomarkers. In contrast, a significant increase in the abundance of *Erysipelotrichaceae* family may serve as risk warning biomarkers, indicating that individuals should enhance the screening and health management efforts (95, 188, 189). This microbiome-based risk assessment is more accurate and individualized than relying solely on clinical indicators.

Endometrial cancer is an estrogen-dependent tumor. Therefore, detecting the abundance of gut microbiota related to estrogen metabolism (risk-related genus *Lachnospira* and the family *Bifidobacteriaceae*) can indirectly reflect an individual’s estrogen recycling levels, thus assessing the potential stimulatory effects on the endometrium. Many identified protective bacteria (certain members of the family *Ruminococcaceae*) are key producers of SCFAs like butyrate. Butyrate has multiple benefits, including regulating blood sugar, anti-inflammatory effects, and inhibiting tumor cell proliferation. Therefore, the levels of these microbiota can serve as indicators of host metabolic health and immune homeostasis, with their reduction potentially signaling a systemic microenvironment conducive to cancer development (189). In summary, microbiome has potential as a biomarker for endometrial cancer that goes beyond simple risk association. It can stratify risk, reveal mechanisms, and guide interventions, providing a scientific basis for achieving unprecedented personalized and population-specific prevention.

#### 5.1.3 Unlocking new dimensions in ovarian cancer diagnosis and treatment: potential biomarkers

Ovarian cancer presents a formidable challenge in the realm of gynecologic malignancies, characterized by a high mortality rate, the complexity of early diagnosis, and a propensity for developing drug resistance. In the ongoing quest for innovative diagnostic and therapeutic strategies, the gut microbiome has emerged as a promising and non-invasive biomarker, distinguished by its myriad advantages. Recent studies have illuminated the unique structural characteristics of the microbiome in ovarian cancer patients, which starkly contrast with those observed in healthy individuals. Notably, there is a discernible increase in the abundance of specific bacterial taxa, such as *Bacteroides*, *Prevotella*, and *Proteobacteria*, while beneficial microorganisms, including *Ruminococcus* and *Actinobacteria*, exhibit a significant decline. This distinctive pattern of microbial dysbiosis functions akin to a microbial fingerprint, offering exciting potential for the development of a non-invasive screening tool aimed at identifying high-risk populations (190).

Individual differences in response to chemotherapy and immunotherapy play a pivotal role in treatment failure and recurrence in ovarian cancer. Platinum-based drugs remain the cornerstone of chemotherapy for this malignancy; however, the emergence of resistance presents a significant challenge. Extensive research has elucidated that the composition of the gut microbiota can profoundly influence the efficacy of platinum-based chemotherapy. Notably, the administration of antibiotics may severely disrupt the delicate balance of the microbiota, potentially leading to accelerated tumor growth and diminished sensitivity to chemotherapy (175, 176, 178). Thus, detecting the gut microbiota of patients before chemotherapy can help assess its potential as a biomarker and predict sensitivity to platinum-based drugs, thereby optimizing treatment regimens. Although immune checkpoint inhibitors (ICIs) have brought hope, their efficacy still needs improvement, and the gut microbiota influences the effectiveness of immunotherapy by modulating the immune system; specific bacteria may serve as biomarkers to predict responders. The dynamic changes in the gut microbiota are closely related to patients’ long-term survival and recurrence risk. A pro-cancer microbiota environment may accelerate tumor growth and result in poorer treatment responses, indicating a worse prognosis. Therefore, specific gut microbiota could serve as independent prognostic factors for assessing long-term survival rates and recurrence risks. Monitoring changes in the fecal microbiota of patients before and after treatment, as well as during recovery, can provide dynamic observations of potential recurrence and resistance. In summary, the gut microbiota plays a crucial role in the occurrence and treatment of ovarian cancer, with the potential to become a multidimensional biomarker, promoting the advancement of diagnosis and treatment towards precision and personalization.

#### 5.1.4 Challenges and future directions: towards robust microbiome-based diagnostics

Despite the transformative potential of microbial biomarkers in reshaping the diagnostic landscape of gynecological malignancies, the translation of these findings from bench to bedside is currently impeded by a “reproducibility crisis.” This challenge stems not from a lack of association, but from the intrinsic complexity and high inter-individual variability of the human microbiota. The composition of the gut and cervicovaginal ecosystems is not a static trait but a dynamic entity, continuously sculpted by a myriad of confounding factors including host genetics, ethnicity, dietary habits, antibiotic usage, and geographic location. Recent large-scale meta-analyses have highlighted that geographic origin often explains a greater proportion of microbial variance than the disease state itself. A pertinent example is provided by a recent Mendelian randomization study on endometrial cancer, which revealed distinct causal associations between specific gut taxa and cancer risk in European populations compared to East Asian populations (189). This underscores a critical limitation: a microbial biosignature identified in a specific demographic cohort may lack the necessary diagnostic sensitivity or specificity when extrapolated to a global population with divergent genetic and environmental backgrounds. A fundamental conceptual hurdle in overcoming this heterogeneity lies in the phenomenon of functional redundancy. In complex microbial communities, distinct bacterial taxa often possess homologous gene clusters capable of performing identical metabolic functions. Consequently, taxonomic variation does not always equate to functional alteration (13, 58). Relying solely on 16S rRNA gene sequencing—which provides a taxonomic census but lacks functional resolution—may obscure the true pathological mechanism. For instance, while the specific bacterial species constituting the “estrobolome” may vary significantly between individuals, the net enzymatic activity of β*-*glucuronidase—which drives estrogen reabsorption and promotes hormone-dependent tumorigenesis—might be the conserved oncogenic driver (33, 36, 132). Thus, the search for universal biomarkers must shift from identifying specific “bacterial names” to detecting conserved functional signatures, such as the enrichment of genes involved in inflammation-inducing LPS biosynthesis or tumor-promoting secondary bile acid metabolism (123, 127). To bridge the chasm between discovery and clinical application, future research paradigms must evolve toward multi-omics integration and systems biology. Emerging investigations in cervical cancer have demonstrated the efficacy of combining metagenomics with metabolomics to reconstruct the “microbe-host interactome.” By correlating microbial gene abundance with the levels of key metabolites—such as nucleotides, amino acids, and lipids—researchers can filter out taxonomic noise and pinpoint the actual metabolic dysfunctions fueling the transition from precancerous lesions to invasive carcinoma (123, 124, 127). Furthermore, integrating host proteomics allows for the assessment of how these microbial metabolic signals are transduced into host immune responses, providing a holistic view of the pathogenic landscape. Finally, to resolve the “chicken-or-egg” causality dilemma—determining whether dysbiosis is a driver of malignancy or a consequence of the TME—robust longitudinal cohort studies are indispensable. Future initiatives must prioritize large-scale, multi-center collaborations utilizing standardized sampling and sequencing protocols to minimize technical batch effects. Coupled with advanced machine learning algorithms capable of integrating high-dimensional microbiome data with clinical metadata, these rigorous approaches are essential for establishing a precise, reproducible, and universally applicable risk stratification system for gynecological cancers.

### 5.2 Dietary interventions: regulation of microbiota composition and metabolite production by high-fiber and Mediterranean diets

Diet is an important external factor for regulating the structure and function of the gut microbiota, and the core of intervention strategies lies in reversing the adverse effects brought by high-fat diets. Excessive fat intake stimulates bile secretion, which is converted by specific microbiota into high concentrations of carcinogenic SBAs. This not only promotes inflammation but also creates a favorable environment for tumor development. In contrast, protective dietary patterns represented by high-fiber and Mediterranean diets exert their anti-cancer effects through multiple mechanisms. These dietary patterns function as prebiotics, promoting the proliferation of beneficial bacteria, which in turn produce key metabolites such as butyrate that possess anti-inflammatory and cancer cell-suppressive properties. Additionally, they help increase fecal bulk, enabling the physical dilution of carcinogenic substances in the intestinal lumen. Furthermore, these dietary habits contribute to maintaining a low-toxicity bile acid pool, collectively preserving the health of the intestinal epithelium and reducing the risk of tumor occurrence (191–193).

### 5.3 Prebiotics, probiotics, and synbiotics: challenges in auxiliary application in cancer treatment

The aim is to actively reshape the gut microecology by directly supplementing beneficial microorganisms (probiotics), their growth substrates (prebiotics), or a combination of both (synbiotics), in order to exert an auxiliary anti-cancer effect (194). Its potential mechanism of action is multifaceted, primarily involving the regulation of gut metabolism and the enhancement of host barrier function (195). Specific probiotics, especially *Lactobacillus* and *Bifidobacterium*, can produce bile salt hydrolase (BSH), which hydrolyzes conjugated bile acids to alter the composition of bile acid pool. This not only potentially affects their toxicity but can also competitively inhibit harmful bacteria from transforming primary bile acids into SBAs that have carcinogenic potential (196, 197). In addition, these interventions can reduce the production of harmful metabolites such as endotoxins by suppressing the growth of pathogenic bacteria, and they can strengthen the intestinal epithelial barrier by upregulating tight junction proteins, thereby limiting the entry of carcinogens and inflammatory factors into the circulatory system (198, 199). However, despite the broad application prospects of these mechanisms, effectively translating them into standard clinical practice still faces significant challenges. Currently, there is a lack of large-scale and rigorously designed clinical trials to confirm their specific efficacy, and their application is restricted by various complex factors, including the highly strain-specific nature of probiotic effects, individual differences caused by host microbiomes and genetic backgrounds, the ability of exogenous strains to establish long-term colonization, and the potential safety risks of using live bacteria in immunocompromised cancer patients.

### 5.4 FMT: exploratory research in reversing chemotherapy resistance and enhancing immunotherapy efficacy

FMT represents a paradigm shift in oncological therapeutics, moving beyond direct tumor targeting to the systemic reconstruction of the host ecosystem. Unlike single-strain probiotics, FMT involves the transfer of a complete, complex microbial community from “complete responders” to refractory patients. The fundamental objective is to reverse the dysbiotic configuration that enforces immunosuppression, thereby re-establishing a homeostatic host-microbe interface capable of supporting systemic anti-tumor immunity. The most robust clinical validation for FMT currently lies in overcoming primary resistance to ICIs. Landmark trials in melanoma and renal cell carcinoma have demonstrated that FMT can successfully convert “non-responders” into “responders.” The underlying mechanism is strictly immunological: successful engraftment of immunostimulatory taxa restores the production of key metabolites such as inosine and butyrate. These signaling molecules act on intestinal DCs to enhance antigen presentation and stimulate the secretion of T-cell-attracting chemokines. This establishes a “gut-tumor axis” that facilitates the trafficking of IFN-γ^+^ CD8^+^ T cells into the tumor bed, effectively transforming an “immune-cold” microenvironment into an inflamed, ICI-responsive phenotype (200–202). In the specific context of gynecological malignancies, particularly ovarian cancer, resistance to platinum-taxane regimens remains a critical bottleneck. Emerging preclinical evidence suggests that FMT serves as a “systemic rescue” strategy to mitigate this resistance. The efficacy of oxaliplatin and cisplatin partially relies on the gut microbiota to prime myeloid cells for ROS production. Antibiotic-induced dysbiosis abrogates this priming effect, whereas FMT restores the “microbial tone” required for chemotherapy to induce effective immunogenic cell death (ICD). Dysbiosis leads to the accumulation of toxic secondary bile acids and systemic endotoxemia, which can upregulate multidrug resistance (MDR) transporters in tumor cells via NF-κB signaling. By replenishing SCFA-producing commensals, FMT repairs the gut epithelial barrier, reduces systemic inflammatory burden, and potentially downregulates these resistance mechanisms (193, 200, 203). Despite this promise, FMT remains an investigational modality in gynecological oncology. Its broad clinical application is currently limited by the intrinsic variability of donor stool (“super-donor” phenomenon) and safety concerns regarding the unintended translocation of multi-drug resistant pathobionts in immunocompromised hosts. Consequently, the field is actively transitioning towards Precision Microbial Therapeutics—defined consortia of bacteria that mimic the functional efficacy of FMT without the biological risks associated with undefined whole-stool transfer. Rigorous RCTs are essential to validate these concepts specifically within the unique immunological landscape of gynecological cancers (92, 204–206).

### 5.5 Precision microbial therapy: designing live biotherapeutics based on specific strains or metabolites

Precision microbial therapy is a new direction in microbiome research, aiming to go beyond traditional FMT by developing highly specialized and standardized live biotherapeutics for precise intervention in the tumor microenvironment (203). This type of therapy primarily includes three levels: First is the specific strain cocktail therapy designed based on an in-depth understanding of microbial functions. By combining strains that efficiently metabolize bile acids, stably produce butyrate, or inhibit pathogenic bacteria, the aim is to achieve a synergistic anti-cancer effect (207). The second is metabolite supplementation therapy, which involves directly oral administration of beneficial metabolite formulations such as butyrate. This approach uses their mechanism as HDACs inhibitors to exert direct anti-cancer activity (208, 209). The third is the most forward-looking engineering bacteria therapy, which utilizes synthetic biology techniques to modify bacteria so that they can respond to tumor microenvironment signals, colonize in tumor sites, and locally release anticancer molecules or detoxifying enzymes, achieving precise strikes (210, 211). The common scientific basis of these advanced therapies lies in the role of the gut microbiome in regulating key metabolites such as bile acids and SCFAs, which play a core role in the occurrence and development of cancer. The ultimate goal of all these intervention strategies is to precisely modulate the gut microecology, transforming a pro-cancer microenvironment into an anti-cancer microenvironment.

## 6 Conclusions

In summation, the microbiome and its metabolic repertoire function as an integral determinant within the pathophysiological architecture of gynecological malignancies. This review elucidates the mechanistic framework underpinning this ecosystem’s influence. First, acting as an immunological rheostat, the microbiome governs the delicate balance between immunosurveillance and inflammation: whereas eubiotic metabolites reinforce mucosal barrier integrity and tolerance, dysbiosis precipitates the translocation of PAMPs, triggering chronic inflammation and recruiting myeloid-derived suppressor cells to subvert antitumor immunity. Second, via direct genotoxicity, specific pathobionts synthesize metabolites that inflict double-strand DNA breaks and disable repair machinery, actively instigating genomic instability—a hallmark of oncogenesis. Third, functioning as a virtual endocrine organ, the “estrobolome” regulates systemic hormonal bioavailability; heightened β-glucuronidase activity drives the enterohepatic recirculation of estrogens, orchestrating a hyperestrogenic microenvironment that fuels hormone-dependent tumorigenesis. The distinctive merit of the synthesis lies in its multidimensional deconstruction of the “gut-reproductive tract axis,” transcending anatomical compartmentalization to integrate disparate evidence into a unified biological model.

By rigorously examining the convergence of metabolic reprogramming, immune modulation, and epigenetic landscapes, we provide a holistic perspective that extends beyond single-organ pathology. Nevertheless, we must critically concede the epistemological limitations inherent to the current landscape. The preponderance of cross-sectional and preclinical data introduces ambiguity in distinguishing causative microbial drivers from secondary consequences of the tumor microenvironment. Moreover, the profound inter-individual heterogeneity driven by host genetics and environmental exposures, compounded by microbial functional redundancy, presents a significant bottleneck in translating taxonomic signatures into standardized clinical utility.

Ultimately, deciphering the molecular interplay within this ecosystem provides a pivotal theoretical basis for understanding the microbial origins of gynecological malignancies. To advance the field, future investigations must prioritize longitudinal, multi-omics cohorts alongside rigorous randomized controlled trials. Such initiatives are indispensable for validating microbiome-modulated interventions—spanning from precision probiotics to fecal microbiota transplantation—catalyzing the shift from correlative observations to the realization of personalized oncological therapeutics.

## Glossary

AhR: Aryl hydrocarbon receptor
BRCA: Breast cancer susceptibility gene
BSH: Bile salt hydrolase
CDC: Cholesterol-dependent cytolysin
CIN: Cervical intraepithelial neoplasia
CST: Community state type
CTLs: Cytotoxic T lymphocytes
DCA: Deoxycholic acid
DCs: Dendritic cells
DDR: DNA damage response
EMMPRIN: Extracellular matrix metalloproteinase inducer
EMT: Epithelial-mesenchymal transition
ERs: Estrogen receptors
FIGO: International Federation of Gynecology and Obstetrics
FMT: Fecal microbiota transplantation
FXR: Farnesoid X receptor
GALT: Gut-associated lymphoid tissue
GPCRs: G protein-coupled receptors
HDACs: Histone deacetylases
Hh: Hedgehog
HPV: Human papillomavirus
HRD: Homologous recombination deficiency
ICD: Immunogenic cell death
ICIs: Immune checkpoint inhibitors
IDO: Indoleamine 2,3-dioxygenase
IELs: Intraepithelial lymphocytes
IP-10: Interferon-inducible protein-10
LPS: Lipopolysaccharide
MAMPs: Microbe-associated molecular patterns
MDR: Multidrug resistance
MDSCs: Myeloid-derived suppressor cells
MIP-1α: Macrophage inflammatory protein-1α
MMP-8: Matrix metalloproteinase-8
NF-κB: Nuclear factor kappa B
NK: Natural killer
PAMPs: Pathogen-associated molecular patterns
PARP: Poly ADP-ribose polymerase
PFS: Progression-free survival
PID: Pelvic inflammatory disease
PRRs: Pattern recognition receptors
RCTs: Randomized controlled trials
ROS: Reactive oxygen species. SBAs, Secondary bile acids
SCFAs: Short-chain fatty acids
TAAs: Tumor-associated antigens
TAMs: Tumor-associated macrophages
Tfh: T follicular helper cells
TGF-β: Transforming growth factor-β
TGR5: G protein-coupled bile acid receptor 1
TIGIT: T cell immunoreceptor with Ig and ITIM domains
TLR4: Toll-like receptor 4
TLS: Tertiary lymphoid structures
TME: Tumor microenvironment
TNF-α: Tumor necrosis factor-α
Tregs: Regulatory T cells
VMT: Vaginal microbiota transplantation.
